# *CmARF3*–*CmTCP7* module regulates flowering time in chrysanthemum (*Chrysanthemum morifolium*)

**DOI:** 10.1093/hr/uhaf095

**Published:** 2025-05-23

**Authors:** Chang Tian, Lisheng Zhai, Jingjing Wang, Wenjing Zhu, Chunmei Shi, Jiafu Jiang, Kunkun Zhao, Fei Li, Lijie Zhou, Aiping Song, Guosheng Xiong, Shengben Li, Fadi Chen, Sumei Chen

**Affiliations:** National Key Laboratory of Crop Genetics & Germplasm Enhancement and Utilization, Nanjing Agricultural University, No. 1 Weigang, Xuanwu District, Nanjing 210095, China; College of Horticulture, Nanjing Agricultural University, Nanjing Agricultural University, No. 1 Weigang, Xuanwu District, Nanjing 210095, China; Key Laboratory of Flower Biology and Germplasm Innovation, Ministry of Agriculture and Rural Affairs, Nanjing Agricultural University, No. 1 Weigang, Xuanwu District, Nanjing 210095, China; Key Laboratory of Biology of Ornamental Plants in East China, National Forestry and Grassland Administration, Zhongshan Biological Breeding Laboratory, Nanjing Agricultural University, No. 1 Weigang, Xuanwu District, Nanjing 210095, China; National Key Laboratory of Crop Genetics & Germplasm Enhancement and Utilization, Nanjing Agricultural University, No. 1 Weigang, Xuanwu District, Nanjing 210095, China; College of Horticulture, Nanjing Agricultural University, Nanjing Agricultural University, No. 1 Weigang, Xuanwu District, Nanjing 210095, China; Key Laboratory of Flower Biology and Germplasm Innovation, Ministry of Agriculture and Rural Affairs, Nanjing Agricultural University, No. 1 Weigang, Xuanwu District, Nanjing 210095, China; Key Laboratory of Biology of Ornamental Plants in East China, National Forestry and Grassland Administration, Zhongshan Biological Breeding Laboratory, Nanjing Agricultural University, No. 1 Weigang, Xuanwu District, Nanjing 210095, China; National Key Laboratory of Crop Genetics & Germplasm Enhancement and Utilization, Nanjing Agricultural University, No. 1 Weigang, Xuanwu District, Nanjing 210095, China; College of Horticulture, Nanjing Agricultural University, Nanjing Agricultural University, No. 1 Weigang, Xuanwu District, Nanjing 210095, China; Key Laboratory of Flower Biology and Germplasm Innovation, Ministry of Agriculture and Rural Affairs, Nanjing Agricultural University, No. 1 Weigang, Xuanwu District, Nanjing 210095, China; Key Laboratory of Biology of Ornamental Plants in East China, National Forestry and Grassland Administration, Zhongshan Biological Breeding Laboratory, Nanjing Agricultural University, No. 1 Weigang, Xuanwu District, Nanjing 210095, China; National Key Laboratory of Crop Genetics & Germplasm Enhancement and Utilization, Nanjing Agricultural University, No. 1 Weigang, Xuanwu District, Nanjing 210095, China; College of Horticulture, Nanjing Agricultural University, Nanjing Agricultural University, No. 1 Weigang, Xuanwu District, Nanjing 210095, China; Key Laboratory of Flower Biology and Germplasm Innovation, Ministry of Agriculture and Rural Affairs, Nanjing Agricultural University, No. 1 Weigang, Xuanwu District, Nanjing 210095, China; Key Laboratory of Biology of Ornamental Plants in East China, National Forestry and Grassland Administration, Zhongshan Biological Breeding Laboratory, Nanjing Agricultural University, No. 1 Weigang, Xuanwu District, Nanjing 210095, China; National Key Laboratory of Crop Genetics & Germplasm Enhancement and Utilization, Nanjing Agricultural University, No. 1 Weigang, Xuanwu District, Nanjing 210095, China; College of Horticulture, Nanjing Agricultural University, Nanjing Agricultural University, No. 1 Weigang, Xuanwu District, Nanjing 210095, China; Key Laboratory of Flower Biology and Germplasm Innovation, Ministry of Agriculture and Rural Affairs, Nanjing Agricultural University, No. 1 Weigang, Xuanwu District, Nanjing 210095, China; Key Laboratory of Biology of Ornamental Plants in East China, National Forestry and Grassland Administration, Zhongshan Biological Breeding Laboratory, Nanjing Agricultural University, No. 1 Weigang, Xuanwu District, Nanjing 210095, China; National Key Laboratory of Crop Genetics & Germplasm Enhancement and Utilization, Nanjing Agricultural University, No. 1 Weigang, Xuanwu District, Nanjing 210095, China; College of Horticulture, Nanjing Agricultural University, Nanjing Agricultural University, No. 1 Weigang, Xuanwu District, Nanjing 210095, China; Key Laboratory of Flower Biology and Germplasm Innovation, Ministry of Agriculture and Rural Affairs, Nanjing Agricultural University, No. 1 Weigang, Xuanwu District, Nanjing 210095, China; Key Laboratory of Biology of Ornamental Plants in East China, National Forestry and Grassland Administration, Zhongshan Biological Breeding Laboratory, Nanjing Agricultural University, No. 1 Weigang, Xuanwu District, Nanjing 210095, China; National Key Laboratory of Crop Genetics & Germplasm Enhancement and Utilization, Nanjing Agricultural University, No. 1 Weigang, Xuanwu District, Nanjing 210095, China; College of Horticulture, Nanjing Agricultural University, Nanjing Agricultural University, No. 1 Weigang, Xuanwu District, Nanjing 210095, China; Key Laboratory of Flower Biology and Germplasm Innovation, Ministry of Agriculture and Rural Affairs, Nanjing Agricultural University, No. 1 Weigang, Xuanwu District, Nanjing 210095, China; Key Laboratory of Biology of Ornamental Plants in East China, National Forestry and Grassland Administration, Zhongshan Biological Breeding Laboratory, Nanjing Agricultural University, No. 1 Weigang, Xuanwu District, Nanjing 210095, China; National Key Laboratory of Crop Genetics & Germplasm Enhancement and Utilization, Nanjing Agricultural University, No. 1 Weigang, Xuanwu District, Nanjing 210095, China; College of Horticulture, Nanjing Agricultural University, Nanjing Agricultural University, No. 1 Weigang, Xuanwu District, Nanjing 210095, China; Key Laboratory of Flower Biology and Germplasm Innovation, Ministry of Agriculture and Rural Affairs, Nanjing Agricultural University, No. 1 Weigang, Xuanwu District, Nanjing 210095, China; Key Laboratory of Biology of Ornamental Plants in East China, National Forestry and Grassland Administration, Zhongshan Biological Breeding Laboratory, Nanjing Agricultural University, No. 1 Weigang, Xuanwu District, Nanjing 210095, China; National Key Laboratory of Crop Genetics & Germplasm Enhancement and Utilization, Nanjing Agricultural University, No. 1 Weigang, Xuanwu District, Nanjing 210095, China; College of Horticulture, Nanjing Agricultural University, Nanjing Agricultural University, No. 1 Weigang, Xuanwu District, Nanjing 210095, China; Key Laboratory of Flower Biology and Germplasm Innovation, Ministry of Agriculture and Rural Affairs, Nanjing Agricultural University, No. 1 Weigang, Xuanwu District, Nanjing 210095, China; Key Laboratory of Biology of Ornamental Plants in East China, National Forestry and Grassland Administration, Zhongshan Biological Breeding Laboratory, Nanjing Agricultural University, No. 1 Weigang, Xuanwu District, Nanjing 210095, China; National Key Laboratory of Crop Genetics & Germplasm Enhancement and Utilization, Nanjing Agricultural University, No. 1 Weigang, Xuanwu District, Nanjing 210095, China; College of Horticulture, Nanjing Agricultural University, Nanjing Agricultural University, No. 1 Weigang, Xuanwu District, Nanjing 210095, China; Key Laboratory of Flower Biology and Germplasm Innovation, Ministry of Agriculture and Rural Affairs, Nanjing Agricultural University, No. 1 Weigang, Xuanwu District, Nanjing 210095, China; Key Laboratory of Biology of Ornamental Plants in East China, National Forestry and Grassland Administration, Zhongshan Biological Breeding Laboratory, Nanjing Agricultural University, No. 1 Weigang, Xuanwu District, Nanjing 210095, China; Plant Phenomics Research Center, Nanjing Agricultural University, No. 1 Weigang, Xuanwu District, Nanjing 210095, China; Plant Phenomics Research Center, Nanjing Agricultural University, No. 1 Weigang, Xuanwu District, Nanjing 210095, China; National Key Laboratory of Crop Genetics & Germplasm Enhancement and Utilization, Nanjing Agricultural University, No. 1 Weigang, Xuanwu District, Nanjing 210095, China; College of Horticulture, Nanjing Agricultural University, Nanjing Agricultural University, No. 1 Weigang, Xuanwu District, Nanjing 210095, China; Key Laboratory of Flower Biology and Germplasm Innovation, Ministry of Agriculture and Rural Affairs, Nanjing Agricultural University, No. 1 Weigang, Xuanwu District, Nanjing 210095, China; Key Laboratory of Biology of Ornamental Plants in East China, National Forestry and Grassland Administration, Zhongshan Biological Breeding Laboratory, Nanjing Agricultural University, No. 1 Weigang, Xuanwu District, Nanjing 210095, China; National Key Laboratory of Crop Genetics & Germplasm Enhancement and Utilization, Nanjing Agricultural University, No. 1 Weigang, Xuanwu District, Nanjing 210095, China; College of Horticulture, Nanjing Agricultural University, Nanjing Agricultural University, No. 1 Weigang, Xuanwu District, Nanjing 210095, China; Key Laboratory of Flower Biology and Germplasm Innovation, Ministry of Agriculture and Rural Affairs, Nanjing Agricultural University, No. 1 Weigang, Xuanwu District, Nanjing 210095, China; Key Laboratory of Biology of Ornamental Plants in East China, National Forestry and Grassland Administration, Zhongshan Biological Breeding Laboratory, Nanjing Agricultural University, No. 1 Weigang, Xuanwu District, Nanjing 210095, China

## Abstract

The precise timing of flowering in response to environment plays a crucial role in the reproductive processes of plants. The FLOWERING LOCUS T (FT)-FD module is a well-established key node in the photoperiod-mediated pathway. However, the identity of novel partners involved in this network and its regulatory mechanisms remain elusive in most nonmodel species. Here, we found that *TEOSINTE BRANCHED1*/*CYCLOIDEA*/*PROLIFERATING CELL FACTOR7* (*CmTCP7*) functions as a floral repressor in *Chrysanthemum morifolium*. Its upstream transcriptional regulator *AUXIN RESPONSE FACTOR3* (*CmARF3*) promotes flowering by directly repressing *CmTCP7* expression. The expression levels of both genes are short-day inducible. Interestingly, FLOWERING LOCUS T-like3 (CmFTL3) interacts with FD-like1 (CmFDL1), which activates flowering*-*accelerating gene *Chrysanthemum Dendrathema MADS111*-*like* (*CmCDM111L*). Meanwhile, CmTCP7 interacts with CmFTL3 and CmFDL1, delaying the CmFTL3 and CmFDL1 complex-promoted flowering in chrysanthemum “Jinba.” These findings reveal a novel regulatory module controlling photoperiod-dependent flowering in chrysanthemum.

## Introduction

Appropriate flowering time is an important adaptive trait for plants to naturally achieve successful sexual reproduction [1, 2]. An unsuitable flowering time can expose plants to harsh environmental conditions and limit the time available for pollination, seed maturation, and dispersal. *Arabidopsis thaliana* is one of the model species for understanding flowering regulation. Multiple flowering mechanisms have been illustrated in this plant, including photoperiod, gibberellin, aging, autonomous, and vernalization pathways [3–5]. Ultimately, these pathways converge on a shared group of downstream regulators of flowering time, including *FLOWERING LOCUS T* (*FT*), *APETALA1* (*AP1*), *SUPPRESSOR OF OVER-EXPRESSION OF CO1* (*SOC1*), and *LEAFY* (*LFY*) [3, 4, 6]. Although the molecular mechanisms regulating flowering time by these key regulators are well understood in *Arabidopsis*, they remain elusive in nonmodel, economically important species.

Several transcription factors (TFs) are involved in flowering [3, 7]. Among these, the TEOSINTE BRANCHED1/CYCLOIDEA/PROLIFERATING CELL FACTOR (TCP) family is a plant-specific group of TFs characterized by a conserved basic helix–loop–helix (bHLH) domain. This family is named after the genes *TEOSINTE BRANCHED1* (*TB1*) from *Zea mays*, *CYCLOIDEA* (*CYC*) from *Antirrhinum majus*, and *PROLIFERATING CELL FACTORS* (*PCF1* and *PCF2*) from *Oryza sativa* [8–10]. In *Arabidopsis*, the *TCP* family consists of 24 members and is classified into classes I and II by the structural characteristics of their TCP domain [11]. The *TCP* genes play a regulatory role in multiple developmental processes, encompassing the differentiation of lateral organs and the formation of axillary meristems [12–14]. Recent studies have demonstrated that *TCPs* play a regulatory role in flowering, particularly in *Arabidopsis*. For example, TCP20 and TCP22, both class I TCPs, interact with the circadian protein LIGHT-REGULATED WD1 (LWD1) to co-activate the *CIRCADIAN CLOCK ASSOCIATED1* (*CCA1*) transcription [15]. Similarly, the promotion of flowering is facilitated by the interaction between Class I TCP7 and nuclear factor-Ys (NF–Ys), wherein this complex directly regulates *SOC1* [16]. TCP15, a class I TCP, positively regulates flowering by directly inducing the expression of the flowering integrator *SOC1* [17]. Other Class I TCPs, such as TCP8 and TCP22, also appear to promote flowering in Arabidopsis [17]. Additionally, Class II TCPs, such as TCP5, TCP13, and TCP17, act as transcriptional activators in *Arabidopsis*. These TCPs interact with FD and further enhance *AP1* expression via FT–FD [18]. TCP4 and other class II CIN-TCPs directly activate *CONSTANS* (*CO*) transcription in leaves [19, 20], the *CO* TF plays a central role in the regulation of photoperiodic flowering. In *Brassica juncea*, the TCP TF *BRANCHED 1* (*BRC1*), induced by short-day (SD) conditions, negatively regulates flowering by directly interacting with the promoters of *BjuFT and BjuFRUITFULL* (*BjuFUL*) [21]. Additionally, in bimolecular fluorescence complementation (BiFC) assays, TCP7 has been observed to interact with FT [22]. However, how this interaction influences flowering remains unclear. Moreover, the reports on *TCP* family genes regulating flowering predominantly focus on the interaction with key factors of flowering or the regulation of downstream flowering genes at the transcriptional level. While the flowering regulation network is well explored, there is still little information about the upstream regulators of *TCP7*.

The auxin response factor (ARF) family is responsible for controlling distinct developmental processes partially by binding to auxin response elements (AuxRE: 5′-TGTCTC-3′) located in the promoters of their target genes. In strawberry (*Fragaria × ananassa*), FaARF4 could directly bind to the promoters of *AP1* and *FUL* and induce the expression of these genes to promote flowering [23]. *ARF3*/*ETTIN* plays critical roles in regulating leaf polarity and reproductive organ patterning [24, 25]. In addition to coordinating growth and patterning, *ARF3* is involved in floral meristem (FM) determinacy through *AGAMOUS* and auxin, which repress cytokinin biosynthesis and signaling. This alters the expression of cell-cycle genes and *WUSCHEL* [26–28]. In apple plants, the WOX4-ARF3-LBD16 module controls plant height by regulating lateral root development [29]. Moreover, *ARF3* is translocated to the organizing center to maintain shoot apical meristem homeostasis by noncell-autonomous means [30]. Overexpression of the *TAS3* ta-siRNA nontargeted *ARF3 mutant* (*ARF3mut*) in *Arabidopsis* promotes an earlier juvenile-to-adult phase transition [26]. However, the mechanisms of how *ARF3* regulates flowering time remain unclear.

For ornamental plants, appropriate flowering timing is critical to ensure maximum yields and a timely supply to the market. Chrysanthemum cultivars, which are primarily SD plants, provide an effective model for analyzing photoperiod-mediated flowering mechanisms. Several studies on flowering have been conducted in chrysanthemums and their closely related species, *Chrysanthemum seticuspe*. In *C. seticuspe*, *CsFTL3* is a key florigen in the photoperiod pathway, which requires SD repeats to gradually induce its expression [31, 32]. *Anti-florigenic FT/TFL1 family protein* (*CsAFT*) and *CsFTL3* have opposite roles in flowering regulation. Flowering occurs primarily when night length exceeds the photosensitive phase for *CsAFT* induction [33]. The basic leucine zipper (bZIP) TFs, CsFDL1, interacts with CsFTL3 and acts as an essential component of the photoperiodic flowering pathway [31, 34, 35]. The interaction between CsFTL3 and CsFDL1 in *C. seticuspe* is essential to promote flowering by activating FM identity genes, including *CsAFL1*, an *AP1*/*FUL*-*like* gene [33, 34]. However, the components involved in FT–FD complex regulated flowering in chrysanthemums remain largely unknown.

We previously studied transcriptional reprogramming of chrysanthemum under SD induction and analyzed the transcriptomes of the apices of chrysanthemum “Jinba.” The apices of plants were collected before and after flower bud differentiation (doming and the involucre differentiation process stage) under a stereo microscope. The changes in well-known flowering time genes such as *CmFTL*, *CmAP1-like*, and *CmAFT* were observed, which suggested the reliability of the transcriptome data. Previous studies have shown that the *TCP* TFs family plays an indispensable role in plant flowering regulation [19, 20]. Interestingly, the expressions of five predicted *TCP* family members, *CmTCP1, CmTCP domain-like protein 1*, *CmTCP2-like*, *CmTCP7*, and *CmTCP15,* were all down-regulated upon SD in chrysanthemum (Supplemental Fig. S1). Several flowering time regulatory genes have been well illustrated, such as *CmTCP15* [17] and *CmTCP2* [20]. Moreover, the abundance of *CmTCP7* was the highest among the *TCP* differentially expressed gene (DEG) members, and its expression level was significantly reduced under SD induction. Previous studies have shown that *AtTCP7* influences flowering time [16]. However, it is unclear whether *CmTCP7* is functionally involved in regulating the flowering process in chrysanthemum. This triggered our curiosity regarding the role of *CmTCP7* in the SD-induced flowering of chrysanthemum.

## Results

### *CmTCP7* delays flowering time of chrysanthemum

**Figure 1:**
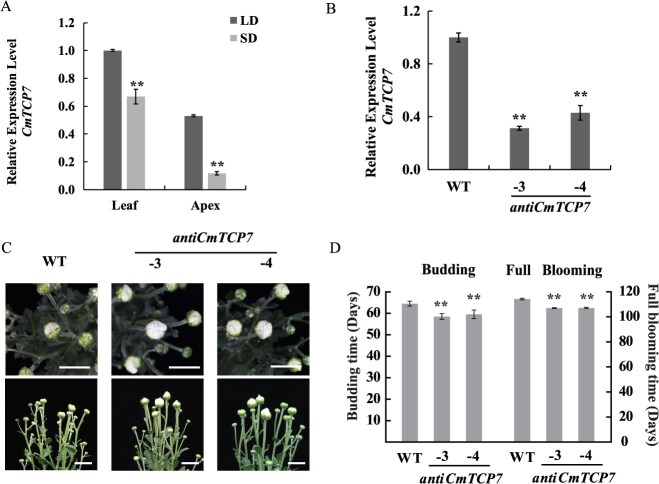
*CmTCP7* suppresses chrysanthemum flowering. (A) Expression levels of *CmTCP7* in WT leaves and apices at vegetative (long-day [LD]) and reproductive stages (3 days after transitioning to short-day [SD] conditions). The values are presented as the mean ± standard deviation (^**^, *P* < 0.01; Student’s *t-*test; *n* = 3). (B) *CmTCP7* expression in *anti-CmTCP7* transgenic plants was determined by real time quantitative PCR (RT-qPCR) and relative to WT. The values are presented as the mean ± standard deviation (^**^, *P* < 0.01; Student’s *t-*test; *n* = 3). (C) Phenotypes of WT and *anti-CmTCP7* transgenic plants. Scale Bars, 2 cm. (D) Statistical comparisons of the flowering time of the budding (BD) and full blooming (FB) stages between the WT and *anti-CmTCP7* transgenic plants. The values are presented as the mean ± standard deviation (^**^, *P* < 0.01; Student’s *t-*test; *n* = 20)

To elucidate the role of *CmTCP7* in chrysanthemum, the 777 bp open reading frame (ORF) of *CmTCP7* was cloned from *C. morifolium* “Jinba.” CmTCP7 shares a highly conserved TCP domain with other members belonging to Class I TCPs (Supplemental Fig. S2). To investigate the potential involvement of *CmTCP7* in SD inducive flowering, we examined its expression levels before and after three-day SD treatment. Our results showed that *CmTCP7* expression was down-regulated by 33% in leaves and 41% in the apices of plants compared to those grown under long-day (LD) conditions (Fig. 1A), suggesting a potential role in the regulation of flowering. However, we did not observe any diurnal rhythm in *CmTCP7* expression over a 48-hour period under either SD or LD conditions, indicating that its expression may not be affected by the circadian clock (Supplemental Fig. S3). Furthermore, we generated a silencing construct using antisense *CmTCP7*, and the regenerated chrysanthemum transformants were designated as *anti-CmTCP7*. The expression level of *CmTCP7* was down-regulated by 68.7% and 57.0% in the *anti-CmTCP7–3* and *−4* transgenic lines compared with that in the wild-type (WT), respectively (Fig. 1B, Supplemental Fig. S4A). Budding time was significantly accelerated by 5–6 days in these transgenic lines, and the full blooming time was significantly accelerated by 7 days compared with those in the WT (Fig. 1C and D). This suggests that *CmTCP7* functions as a flowering time repressor in the chrysanthemum “Jinba.” However, the mechanism by which transcription of *CmTCP7* was regulated remains unclear.

### CmARF3 directly binds to the promoter of *CmTCP7* and represses its transcription

**Figure 2:**
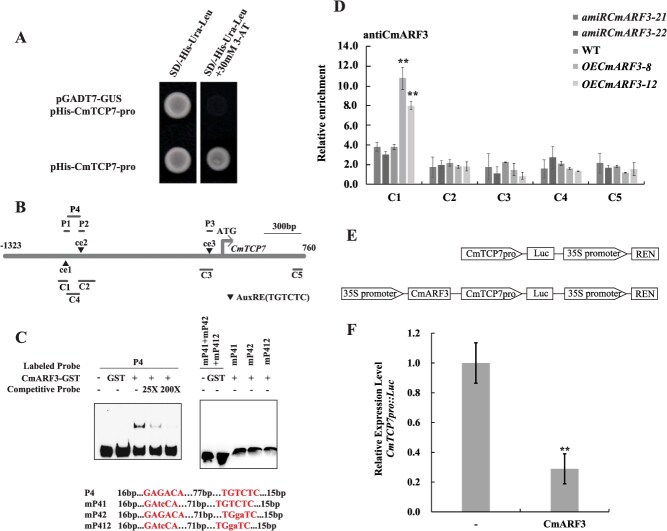
CmARF3 binds directly to the promoter of *CmTCP7* and downregulates *CmTCP7* expression. (A) CmARF3 binds to the promoter of *CmTCP7*, as revealed by a yeast one-hybrid assay. pGADT7-GUS/pHis-CmTCP7-pro were transformed and served as a negative control. (B) The promoter structure of the *CmTCP7* gene and probe fragments used in the EMSA and ChIP assay. The black triangle indicates the position of the AuxREs, which are named ce1, ce2, and ce3, respectively. P1-P4 fragments are used for EMSA and C1-C5 for the ChIP-qPCR assay. (C) EMSA analysis of the binding of recombinant CmARF3 proteins to the P4 probe of the *CmTCP7* promoter, which contains the first two TGTCTC AuxRE sites ce1 and ce2. Sequences of the EMSA probes: capital letters represent TGTCTC AuxRE elements, while lowercase letters represent mutation sites. The oligonucleotides (P4 and P41/42/412) were used as probes; mP41/42/412 represents mutant probes, where ce1, ce2, or both ce1 and ce2 were mutated. The nonlabeled competitive probe was used as a cold probe. Untreated glutathione-s-transferase (GST) protein isolates were used as a control. (D) ChIP-qPCR analysis of the relative binding of CmARF3 to the promoter of *CmTCP7*. ChIP assays were performed with chromatin prepared from WT, two *OECmARF3*, and two *amiRCmARF3* plants using the CmARF3-specific antibody. C5 was used as a control. The values are presented as the mean ± standard deviation (^**^, *P* < 0.01; Student’s *t*-test; *n* = 3). (E) Structure of *CmTCP7pro*:*Luc* and CmARF3_*CmTCP7pro*:*Luc* used in the transient transformation dual-luciferase assays. REN, renilla luciferase. Luc, luciferase. (F) CmARF3 suppresses *CmTCP7* expression in chrysanthemum protoplast cells. The construct of *CmTCP7pro*:*Luc* was used as a control (shown as -). The construct of CmARF3_*CmTCP7pro*:*Luc* was used as the effector (shown as CmARF3). The values are presented as the mean ± standard deviation (^**^, *P* < 0.01; Student’s *t-*test; *n* = 3)

SD induction leads to the down-regulation of *CmTCP7* expression, which suggests that there might be an upstream TF suppressing *CmTCP7* expression. To further explore the upstream transcriptional regulator of *CmTCP7*, we cloned a genomic fragment of 1323 nucleotides prior to the transcriptional initiation site of *CmTCP7*. Bioinformatical prediction of the *cis*-elements using the PLACE website on promoter analysis found that the *CmTCP7* promoter has multiple *cis*-elements associated with hormone signaling pathways (Supplemental Table S1). Additionally, most of these *cis*-elements were involved in auxin signal transduction, including the auxin response elements AuxRE (5′-TGTCTC-3′) [27], which trigger us to hypothesize that CmARF may directly bind to the *CmTCP7* promoter. This inspired us to screen the candidate upstream TF using three tandem copies of the 5′-TGTCTC-3′ construct in a yeast one-hybrid assay (Supplemental Table S2), where the TF CmARF3 was screened out. Sequence alignment and neighbor-joining phylogenetic tree analyses show that CmARF3 is an orthologue of ARF3/ETTIN (Supplemental Fig. S5). Subsequently, the possible binding of TF CmARF3 to the promoter of *CmTCP7* was identified by a yeast one-hybrid assay (Fig. 2A). To confirm the direct binding of CmARF3 to the *CmTCP7* promoter *in vitro*, we expressed the CmARF3-GST recombinant protein in *Escherichia coli*. Moreover, we performed an electrophoretic mobility shift assay (EMSA) with three probes, which were designed corresponding to each AuxRE (Fig. 2B). However, we could not detect direct binding between the CmARF3 protein and each of the three probes (Supplemental Fig. S6A). Previous studies indicated that the palindromic structure composed of two inverted repeat elements in a DNA fragment is helpful for protein binding *in vitro* [36–38]. In the *CmTCP7* promoter, the first two AuxREs are in opposite directions and closely spaced, forming a palindromic structure (Fig. 2B). To test whether the structure formed by the first two AuxREs is critical for protein binding, we designed the P4 probe, which included both AuxREs *cis*-elements and the sequence between them (Fig. 2B). When biotin-labeled P4 was incubated with CmARF3 protein, a clear band was detected, and the band intensities faded with the addition of an unlabeled probe (Fig. 2C). Specific binding was further tested with the mutant probes mP41, mP42, and mP412. As expected, the CmARF3 protein failed to bind to the mutant probes *in vitro*. The results demonstrated that the CmARF3 protein directly and specifically binds to the P4 region of the *CmTCP7* promoter *in vitro*, and the palindrome structure is necessary for stable binding.

**Figure 3:**
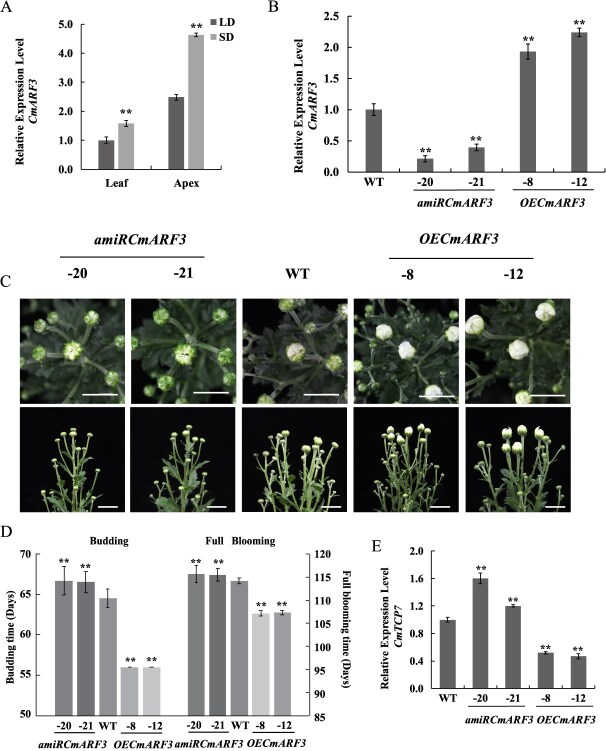
*CmARF3* promotes chrysanthemum flowering. (A) Expression analysis of *CmARF3* in WT leaves and apices at vegetative stage (long-day [LD]) and reproductive stage (3 days after transitioning to short-day [SD] conditions). The values are presented as the mean ± standard deviation (^**^, *P* < 0.01; Student’s *t-*test; *n* = 3). (B) *CmARF3* expression in *amiRCmARF3* and *OECmARF3* transgenic plants as determined by real time quantitative PCR (RT-qPCR) and relative to WT. The values are presented as the mean ± standard deviation (^**^, *P* < 0.01; Student’s *t-*test; *n* = 3). (C) Phenotypes of WT, *OECmARF3,* and *amiRCmARF3* transgenic plants. Scale Bars, 2 cm. (D) Days of the budding time and full blooming time stage of WT, *OECmARF3,* and *amiRCmARF3* transgenic plants. The values are presented as the mean ± standard deviation (^**^, *P* < 0.01; Student’s *t-*test; *n* = 20). (E) *CmTCP7* expression in *amiRCmARF3* and *OECmARF3* transgenic plants as determined by RT-qPCR and relative to WT. The values are presented as the mean ± standard deviation (^**^, *P* < 0.01; Student’s *t-*test; *n* = 3)

To investigate the *in vivo* interaction between CmARF3 and the *CmTCP7* promoter, we employed a chromatin immunoprecipitation (ChIP) assay in conjunction with qPCR. First, we generated *amiRCmARF3* plants using artificial microRNA (amiRNA) technology to specifically silence the *CmARF3* gene, as well as *OECmARF3* plants by overexpressing *CmARF3* in chrysanthemum (Fig. 3B, Supplemental Fig. S4B). The efficiency and specificity of the CmARF3 antibody were validated through western blotting in both the *OECmARF3* and *amiRCmARF3* transgenic lines. Western blotting results demonstrated that the *OECmARF3* plants had a higher CmARF3 accumulation level than that in the WT, whereas the *amiRCmARF3* plants had the lowest accumulation levels (Supplemental Figs S6B and S7B). Next, we compared the enrichment of CmARF3 on the *cis*-elements of *CmTCP7* among the WT, *OECmARF3,* and *amiRCmARF3* plants. Five pairs of primers were used to amplify the fragments C1, C2, C3, and C4, which flank the P1, P2, P3, and P4 fragments in the *CmTCP7* promoter, respectively. The C5 fragment was employed as a negative control (Fig. 2B). In line with our EMSA assay, the ChIP-qPCR assay showed no enrichment in the C2 and C3 regions compared with that in the control (Fig. 2D). In contrast, significant enrichment was detected around the C1 region, which harbors the *cis*-element ce1 alone, with more significant enrichments detected in the C1 region in *OECmARF3* transgenic plants compared with that in the WT. This indicates that the CmARF3 protein specifically binds to the ce1 AuxRE *cis*-element of the *CmTCP7* promoter *in vivo* (Fig. 2D, Supplemental Fig. S7).

Various ARFs have been reported to activate or repress the expression of downstream genes [27, 28]. This prompted us to ask how CmARF3 affects *CmTCP7* expression. To address this question, plasmids harboring luciferase (Luc) driven by the *CmTCP7* promoter with or without *CmARF3* were co-transfected into chrysanthemum protoplasts prepared from WT leaves and subjected to dual-luciferase assays (Fig. 2E). We found that the protoplasts expressing *CmARF3* significantly inhibited *Luc* expression driven by *CmTCP7pro* compared with the protoplasts expressing *CmTCP7pro:Luc* alone (Fig. 2F). This suggests that CmARF3 can bind the *CmTCP7* promoter directly and inhibit the expression of *CmTCP7 in vivo*.

### CmARF3 promotes early flowering in *C. morifolium* by repressing *CmTCP7* expression

Our results indicate that *CmTCP7* is a direct downstream gene of CmARF3, which triggers us to hypothesize that *CmARF3* may be functionally involved in flowering time regulation. We tested the diurnal expression of *CmARF3* for at least 48 h under SD vs LD conditions. The expression of *CmARF3* did not demonstrate a distinct diurnal rhythm, but levels were generally higher during SD than during LD (Supplemental Fig. S3). If *CmARF3* can potentially regulate flowering, it should be responsive to SD induction. We transferred *C. morifolium* “Jinba” plants grown in LD to SD treatment for three days to ensure that the plants entered the initial stage of flower bud differentiation. Accordingly, *CmARF3* was found to be up-regulated both in the leaves and apices after SD induction (Fig. 3A).

To further identify the function of *CmARF3* in regulating flowering time in chrysanthemum, we monitored flowering time in *amiRCmARF3* plants and *OECmARF3* plants. In the *OECmARF3* lines, flowering time was significantly accelerated. Flower buds emerged 8–9 days earlier, and full bloom occurred 7–8 days earlier than in WT plants. On the other hand, in the *amiRCmARF3* lines, the budding time was delayed by 2–3 days, and blooming occurred 1–2 days later compared to the WT (Fig. 3C and D; Supplemental Fig. S6C). Since CmARF3 directly binds to the *CmTCP7* promoter both *in vivo* and *in vitro* to repress its expression level, we expected that the expression of *CmTCP7* would be affected by CmARF3. To test this, we analyzed the expression levels of *CmTCP7* in *amiRCmARF3* and *OECmARF3* transgenic plants. Compared with WT, we found that *CmTCP7* was up-regulated in the *amiRCmARF3* transgenic plants and down-regulated in the *OECmARF3* transgenic plants (Fig. 3E).

To test whether *CmARF3*-mediated flowering is dependent on *CmTCP7*, we transiently silenced the expression of the *CmTCP7* gene in the *amiRCmARF3* transgenic lines using a virus-based miRNA expression system [39]. Three independent transgenic lines of *amiRCmARF3–22/CalCuv–CmTCP7* were obtained. The expression of *CmTCP7* was down-regulated by 33% in the *amiRCmARF3–22/CalCuv–CmTCP7* lines compared to that in the *amiRCmARF3–22/CalCuv* lines (empty vector infested control). Additionally, the budding time was 5 days earlier compared to that in the *amiRCmARF3–22/CalCuv* line, implying that the regulatory role of *CmARF3* in flowering is dependent on *CmTCP7* (Supplemental Fig. S8). However, how *CmTCP7* regulates flowering remains to be elucidated.

### CmTCP7 interacts with the CmFTL3–CmFDL1 complex to repress the FM identity gene *CmCDM111L* in *C. morifolium*

**Figure 4:**
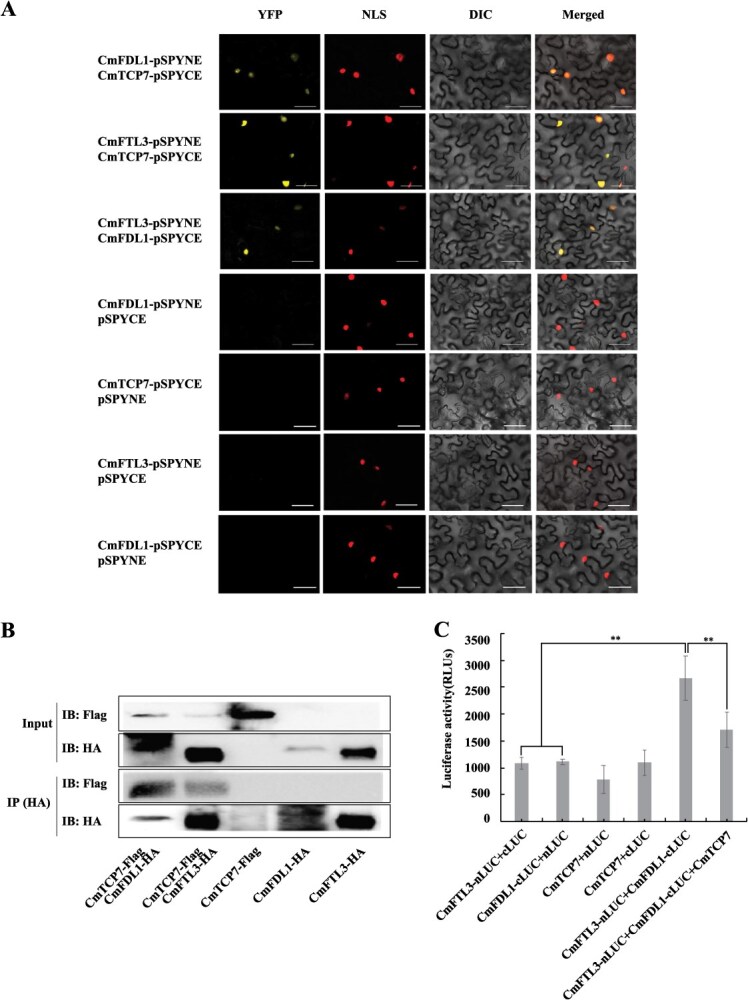
The CmTCP7–CmFTL3–CmFDL1 complex. (A) BiFC assays for CmTCP7, CmFDL1, and CmFTL3. D53-mCherry was used as a nuclear-localized marker (NLS-mcherry). In addition, the pSPYNE and pSPYCE empty vectors were used as negative controls. Scale Bars, 50 μm. (B) Co-IP assays for CmTCP7, CmFDL1, and CmFTL3 interactions. HA-CmFDL1, HA-CmFTL3, and Flag-TCP7 immunoprecipitated complexes were detected in the Co-IP analysis results. Immunoprecipitation was performed using the anti-HA affinity matrix, and immunoaffinity purification of tagged proteins was performed with the HA peptide. HA-CmFDL1 and HA-CmFTL3 were detected using anti-HA high affinity antibody, and co-immunoprecipitated flag-TCP7 was then detected using an antiflag antibody. (C) Firefly luciferase complementation assay in *Nicotiana benthamiana*. The N-terminal half of luciferase (nluc) was fused with CmFTL3, and the C-terminal half of luciferase (cluc) was fused with CmFDL1. The Agrobacteria injection volume was mixed in a ratio of 1:1:3 in the CmFTL3–CmFDL1–CmTCP7 combination. CmTCP7-HA construction was used to express CmTCP7 in this assay. RLUs, Relative Light Units. The values are presented as the mean ± standard deviation (^**^, *P* < 0.01; Student’s *t-*test; *n* = 3)

In *Arabidopsis*, yeast two-hybrid assay (Y2H) and BiFC showed that TCP7 interacts with FT [22]. FT and bZIP TF FD can form heterodimers, further promoting flowering by regulating flowering-related genes [35, 40]. In previous reports, CsFTL3 and CsFDL1 interaction has been demonstrated in *C. seticuspe*, a closely related wild species of *C. morifolium* [34], the CsFTL3–CsFDL1 complex promotes flowering under SD conditions by positively regulating the expression of *CsFTL3* through a feedback mechanism [34]. We hypothesize that CmTCP7 may function as a protein-interacting partner of the FT–FD complex. To test this hypothesis, we first conducted a BiFC assay, where CmTCP7 interacts with both CmFTL3 and CmFDL1 (Fig. 4A). To further validate the interactions *in vivo*, we performed co-immunoprecipitation (Co-IP) assays. CmTCP7-Flag was transiently co-expressed with CmFDL1-HA or CmFTL3-HA in *Nicotiana benthamiana* leaves. Both CmFDL1 and CmFTL3 pulled down the CmTCP7 protein (Fig. 4B), confirming that CmTCP7 could interact with CmFDL1 and CmFTL3 *in vivo*. The *in vivo* interaction was further confirmed via Co-IP assay in chrysanthemum protoplasts (Supplemental Fig. S9). The luciferase complementation assay demonstrated that CmFTL3 and CmFDL1 interacted with each other, and noteworthy, CmTCP7 disturbed the interaction between CmFTL3 and CmFDL1 (Fig. 4C); however, how CmTCP7 disturbs the interaction remained unknown.

**Figure 5:**
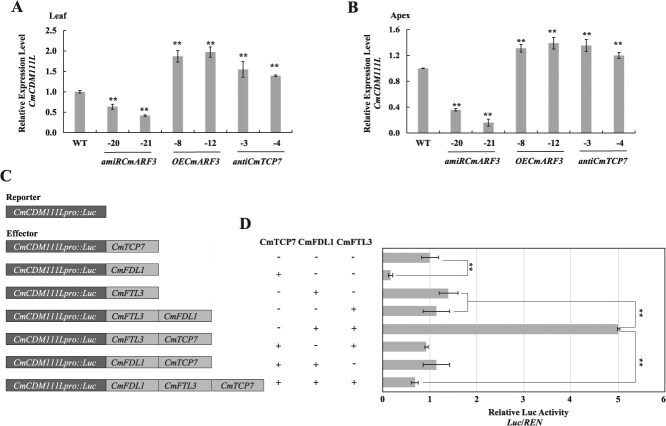
*CmCDM111L* is regulated by the *CmARF3–CmTCP7* transcription cascade. Expression levels of *CmCDM111L* in the leaves (A) and apices (B) of WT, *amiRCmARF3*, *OECmARF3,* and *anti-CmTCP7* transgenic plants under short-day (SD). The values are presented as the mean ± standard deviation (^**^, *P* < 0.01; Student’s *t-*test; *n* = 3). (C) Schematic representation of the reporter and effectors used in the transient transactivation assays. (D) Transient dual-luciferase effectors CmFTL3–CmFDL1 induced the expression of *CmCDM111L,* which was partially antagonized by CmTCP7. The values are presented as the mean ± standard deviation (^**^, *P* < 0.01; Student’s *t-*test; *n* = 3)

The interaction between FT and FD, which is highly conserved, regulates *AP1* expression to promote FM identity during floral transition [35, 41]. In *C. seticuspe*, co-expression of CsFTL3–CsFDL1 in the protoplast significantly induced the expression of *AP1/FUL-like* genes (*CsAFL1* and *CsAFL2*) [33]. The flowering functions of *CsAFL1* and *CsAFL2* have not yet been identified in *C. morifolium* “Jinba.” We previously found that *CmCDM111L* (*CmCDM111-like*, previously designated as *CmAP1L1* and an orthologue of *Arabidopsis AP1*) promotes flowering in *C. morifolium* “Jinba” [42]. In the present study, *CmCDM111L* is significantly up-regulated in *OECmARF3* transgenic plants and down-regulated in *amiRCmARF3* transgenic plants compared with that in WT (Fig. 5A and B). Accordingly, *CmCDM111L* is also up-regulated in the *anti-CmTCP7* transgenic plants (Fig. 5A and B). However, whether the expression of *CmCDM111L* is regulated by the CmFTL3–CmFDL1 interaction in *C. morifolium* and how *CmTCP7* affects this regulation is still unclear. To address this question, dual-luciferase assays were performed on chrysanthemum protoplasts (Fig. 5C). *CmCDM111Lpro:Luc* was transiently expressed in chrysanthemum protoplasts. Neither CmFTL3 nor CmFDL1 alone altered the expression of *CmCDM111L*, but the addition of both proteins significantly increased *Luc* activity, suggesting that the CmFTL3–CmFDL1 interaction is necessary to activate *CmCDM111L* (Fig. 5D). Furthermore, when CmTCP7, CmFTL3, and CmFDL1 were co-transfected into protoplasts, *luc* activity decreased by 80% compared with activity promoted by CmFTL3 and CmFDL1 co-transfection (Fig. 5D). In contrast, the combination of CmTCP7–CmFTL3 and CmTCP7–CmFDL1 had minimal effects on the expression of *CmCDM111L*. Overall, it suggests that CmTCP7 interacts with CmFTL3 and CmFDL1 to antagonize the activating role of the CmFTL3–CmFDL1 complex on *CmCDM111L* expression.

**Figure 6:**
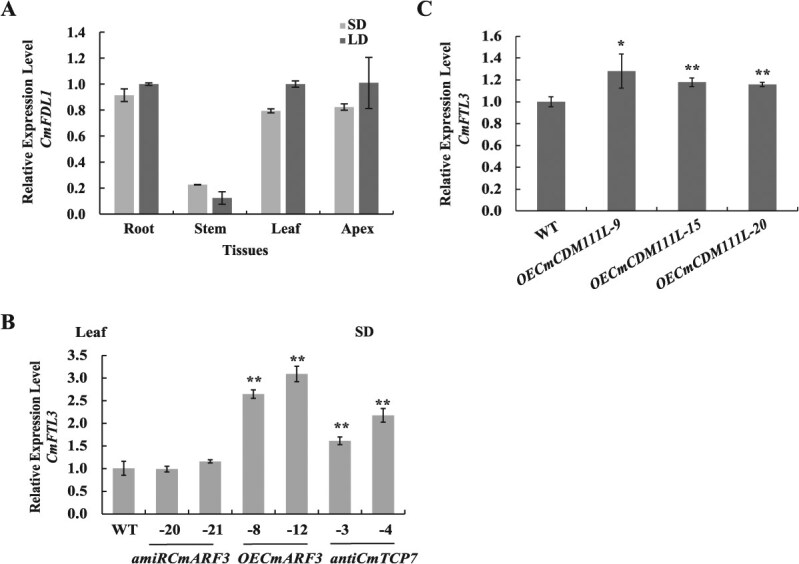
Expression patterns of flowering-time genes in *Chrysanthemum morifolium*. (A) Expression analysis of *CmFDL1* in WT root, stem, leaves, and apices under a long-day (LD) or a short-day (SD) condition. The values are presented as the mean ± standard deviation; *n* = 3. (B) Upregulation of *CmFTL3* by the CmTCP7–CmFDL1–CmFTL3 complex. *CmFTL3* expression in *amiRCmARF3*, *OECmARF3,* and *anti-CmTCP7* transgenic lines under SD treatment as determined by RT-qPCR and relative to WT. The values are presented as the mean ± standard deviation (^*^, *P* < 0.05; ^**^, *P* < 0.01; Student’s *t-*test; *n* = 3). (C) Upregulation of *CmFTL3* in *CmCDM111L-*overexpressed transgenic plants. *CmFTL3* expression in *OECmCDM111L* transgenic lines under natural SD treatment as determined by RT-qPCR and relative to WT. The values are presented as the mean ± standard deviation (^**^, *P* < 0.01; Student’s *t-*test; *n* = 3)

Previous studies have indicated that FT exhibits systemic transportability, and tissues, where *FD* is presented, are potential tissues for the formation of FD–FT complex [35]. Therefore, we detected the expression of *CmFDL1* in different tissues of chrysanthemum “Jinba,” and found that *CmFDL1* was expressed in both the leaves and apices under LD and SD conditions (Fig. 6A), similar to its expression in *C. seticuspe* [33]. Additionally, we observed that *CmFTL3* expression was higher in *OECmARF3* and *anti-CmTCP7* lines compared to that in WT (Fig. 6B). Furthermore, *CmFTL3* expression was upregulated in *CmCDM111L* overexpression transgenic chrysanthemum “Jinba” plants (Fig. 6C). This suggests a potential reciprocal regulatory relationship between *CmFTL3* and *CmCDM111L*, where CmFTL3 might positively regulate *CmCDM111L* expression and *vice versa*.

## Discussion

### CmARF3–*CmTCP7* is a novel transcriptional module involved in SD inducive flowering of chrysanthemum

The regulation of flowering time is a complex process involving multiple pathways that converge to regulate a core set of functionally conserved genes [3]. Several TCP TFs have been shown to play roles in regulating flowering time in *Arabidopsis* [18–20], but the upstream regulatory mechanisms of *TCP* genes remain poorly understood. In this study, we found that SD conditions suppress *CmTCP7* expression. Moreover, we identified CmARF3 as an upstream regulator of *CmTCP7*, providing new insight into the transcriptional regulation of *TCP* genes. Both *in vitro* and *in vivo* evidences verified direct binding of CmARF3 to the *CmTCP7* promoter. Previous studies have indicated that the palindromic structure or composite structure of auxin response elements, and protein–protein interactions, may determine the activity and targeting of ARF3 [36, 43]. In present study, specifically, the palindrome formed by two adjacent elements ce1 and ce2 could facilitate stable binding to CmARF3 protein *in vitro* (Fig. 2B and C). However, *in vivo*, a ChIP-qPCR assay showed high CmARF3 occupancy at C1 (−871 to −767 bp) in the *CmTCP7* promoter in contrast to C4 (−791 to −663 bp) (Fig. 2D). We hypothesize that the marked enrichment of ce1 *in vivo* may result from an interaction between CmARF3 and unidentified proteins. Additionally, we propose that the flanking sequence of element ce1 may have a composite structure, such as a CCTCG motif (Supplemental Fig. S10). Though further experiments would be needed to determine these details.

*ARF3* has previously been shown to play a role in plant development, but in this study, we provide the evidence that *CmARF3* also regulates flowering time in chrysanthemums, highlighting a novel function of this TF. Notably, only a slight delay in full flowering time was observed in *amiRCmARF3* plants. Moreover, these plants showed no significant reduction in *CmFTL3* expression (Fig. 6B), suggesting that the functional redundancy of other *ARFs* with *CmARF3* cannot be ruled out. In *Arabidopsis*, *ARF3,* as well as *ARF2* and *ARF4,* are targets of the miR390-tasiRNA-ARF pathway. *arf2–6*, *arf2–7*, and *arf2–8* mutants display a pleiotropic phenotype, including late flowering under LD conditions, which suggests that *ARF2* is also involved in flowering [44]. Investigating whether other *ARFs* in chrysanthemum contribute to flowering regulation will be an interesting direction for future research.

### *CmARF3* and *CmTCP7* regulates SD inducive flowering of chrysanthemum

The timing of flowering in plants is regulated by the collaborative interplay between environmental signals and endogenous gene networks, with photoperiodic signals playing a pivotal role in the flowering process of many species [45]. Based on their response to daylength, plants can be classified into LD plants and SD plants, which induce flowering when the daylength exceeds or falls below a certain critical threshold, respectively [46]. The transmission of photoperiodic signals relies on light receptors that sense environmental light information and subsequently regulate the expression of flowering-related genes through circadian rhythm [47]. However, the role of orthologues may differ across species with distinct photoperiodic requirements. For example, *CO* enhances responsiveness to photoperiod by accelerating flowering during LD conditions but inhibits flowering under SD conditions [48]. Similarly, *phyC* delays flowering in SD photoperiods but promotes flowering in the absence of *phyA* under LD photoperiods [49]. *C. morifolium*, a typical SD plant, exhibits a complex photoperiodic regulation of flowering. We observed that SD conditions suppress the expression of *CmTCP7* and promotes flowering of chrysanthemum, heterologous overexpression of *CmTCP7* delayed flowering times in *Arabidopsis* (Supplemental Fig. S4D and E). In contrast, its orthologue, *AtTCP7*, promotes flowering in *Arabidopsis* [16]. This difference in the function of the *TCP7* orthologues across species could be attributed to differences in photoperiodic responses. Although orthologues may be involved in flowering control, their functions can diverge significantly depending on the photoperiodic needs and the specific regulatory networks in each plant species. This comparison provides new insights into how orthologues regulate flowering in species with different photoperiodic responses.

ARF3 is a key TF in the auxin-signaling pathway, with most studies on its involvement in gynoecium morphogenesis [25]. However, the mechanism by which ARF3 regulates flowering in SD plants remains unclear. A typical example of flowering induction through auxin is in pineapple, where naphthaleneacetic acid (NAA) treatment leads to ethylene production within one day, and ethylene subsequently promotes flowering [50]. Similarly, the spraying of NAA on litchi and of 2,4-dichlorophenoxyacetic acid (2,4-D) on sweet potato have been shown to induce flowering in both species [51]. In contrast, 2,4-D application on *Citrus unshiu* inhibited flowering [52]. These reports highlight the role of auxin in regulating flowering time depends on the plant species. Given that ARF3 is a key TF in the auxin-signaling pathway in *Arabidopsis*, we sought to test if auxin affects flowering time in chrysanthemums. We treated chrysanthemum “Jinba” with indole-3-acetic acid (IAA) and the PEO–IAA, an auxin antagonist that binds to transport inhibitor response 1/auxin signaling F-box proteins (TIR1/AFBs). However, neither IAA nor PEO–IAA treatment affected the flowering time of chrysanthemum “Jinba” (Supplemental Fig. S11), suggested that *CmARF3* regulated flowering is independent of auxin signaling. Noteworthy, the expression levels of *CmTCP7* or *CmARF3* did not exhibit any diurnal rhythm over a 48-hour period under both SD and LD conditions (Supplemental Fig. S3). However, it was found that both *CmTCP7* and *CmARF3* are responsive to SD induction (Figs 1A and 3A). The results suggest that *CmTCP7* and *CmARF3* may be involved in photoperiod-induced flowering rather than being directly involved in photoperiodic perception. These findings highlight *CmARF3*’s role in the photoperiodic regulation of flowering time, offering new insights into how day length influences floral induction.

### The *ARF3*-*TCP7* transcription cascade regulates the onset and advancement of floral initiation through the flowering pathway controlled by FT–FD.

The FT–FD interaction has been described in several plants displaying divergent flowering photoperiods, including LD species, such as *Arabidopsis* [35], pea [53], kiwifruit [54], wheat [55], and barley [56], and SD species include *C. seticuspe* [33], potato [57], soybean [58], rice [59], *Artemisia annua* [60], and day-neutral species, such as tomato [61] and *Nicotiana tabacum* [62]. The physical interaction between CmFTL3 and CmFDL1 in *C. morifolium* “Jinba” suggests that the FT–FD flowering pathway is probably conserved across different chrysanthemum species.

To verify the functional role of the FT–FD complex, expression of downstream genes, such as *AP1* orthologue, is well assessed [18, 33, 35, 63]. In rice, the 14-3-3 proteins interact with Hd3a (FT homolog) and OsFD1, inducing the transcription of *AP1* orthologue, which leads to flowering [59]. Similarly, In *C. seticuspe*, the CsFDL1 and CsFTL3 complex-induced *AP1* expression, which is antagonized by the suppressor *CsAFT* [33]. In *C. morifolium* “Jinba,” the CmFTL3–CmFDL1 complex influences the expression of *CmCDM111L*, an *AP1* orthologue [42], suggesting a similar regulatory mechanism. The downregulation of *CmCDM111L* in the apices and leaves of *amiRCmARF3* lines, alongside its upregulation in *OECmARF3* lines and *anti-CmTCP7* lines (Fig. 5A and B), suggests that the transcriptional cascade of *CmARF3*, *CmTCP7,* and *CmCDM111L* might be present in both the leaves and apices in “Jinba.” Furthermore, the expression levels of *CsFTL3* showed a close correspondence with the onset of flowering [31]. Previous studies have indicated that the positive feedback regulation of *CsFTL3* might involve *MADS-box* genes, specifically those regulated by CsFTL3–CsFDL1, such as *CsAFL1/2* [33, 34]. Similarly, we observed a positive feedback regulation of *CmFTL3* by *CmCDM111L* (Fig. 6B and C). Such bidirectional regulation could form a positive feedback loop, potentially promoting flowering under continuous SD conditions by enhancing the expression of flowering-related genes and facilitating the plant’s adaptation to environmental cues. However, further experiments are necessary to confirm this hypothesis.

**Figure 7:**
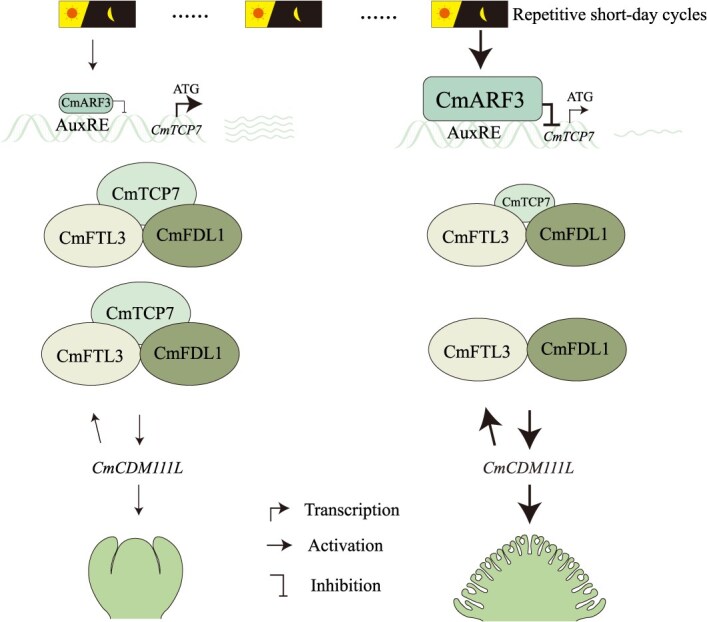
*CmARF3–CmTCP7* model for promoting flowering in chrysanthemum “Jinba.” As short-day cycles accumulate, the expression of *CmARF3* gradually increases, leading to the suppression of *CmTCP7*. This suppression weakens the interaction between CmTCP7 and CmFTL3/CmFDL1. Consequently, the CmFTL3–CmFDL1 complex can further promote the expression of *CmCDM111L*. In turn, CmCDM111L feedbacks to enhance *CmFTL3* expression, forming a positive regulatory loop that reinforces floral induction under short-day conditions, ultimately accelerating flowering in chrysanthemum “Jinba.” The meaning of the arrows is as shown in the figure, with thicker arrows indicating stronger promoting or inhibiting effects

Prolonged exposure to SD conditions leads to increased *CmARF3* expression, which represses *CmTCP7*, thereby alleviating the antagonistic effect on the CmFTL3–CmFDL1 complex-induced expression of *CmCDM111L*, ultimately promoting chrysanthemum flowering (Fig. 7). Furthermore, CmTCP7 functions as a repressor. It is possible that the expression levels of *CmTCP7* could be a rate-limiting factor for CmFTL3–CmFDL1 mediated induction of *CmCDM111L* expression. Co-expression of CmTCP7 seems to partially inhibit the CmFTL3–CmFDL1 interaction based on LUC activity assay (Fig. 4C). However, further research is required to understand the precise impact of CmTCP7 on the CmFTL3–CmFDL1 interaction.

In summary, the suppressor *CmTCP7* participates in modulating flowering at least partially via the CmFTL3–CmFDL1 targeted *CmCDM111L* flowering pathway (Fig. 7). It is noteworthy that in *Arabidopsis*, Class II TCPs such as TCP5/13/17 have been found to interact with FD and enhance the expression of *AP1* [18]. The present study has identified a new member of the *TCP* family that regulates flowering via the FT–FD protein complex. Chrysanthemums are known for their remarkable diversity, with numerous cultivars exhibiting significant variation in flowering time, which is a key trait for both ornamental and agricultural purposes. However, due to the allopolyploid nature of chrysanthemums and the lack of complete genomic resources for many cultivars, investigating the allelic variation of *CmTCP7* across different varieties and its association with flowering time remains challenging. Further genomic studies and the generation of reference genomes for different cultivars will be essential to address this gap and explore the full potential of *CmTCP7* in regulating flowering across chrysanthemum varieties.

## Materials and methods

### Plant materials and growth conditions

The *C. morifolium* “Jinba” cultivar was obtained from the Chrysanthemum Germplasm Resource Preserving Center at Nanjing Agricultural University, located in Nanjing, China. Cuttings from both WT and transgenic plants underwent low temperatures and were rooted under LD conditions (16 hours of light and 8 hours of darkness, 25/22°C, 40% relative humidity). Rooted cuttings were grown under LD conditions until they reached the stage of 14 fully expanded leaves. Subsequently, the plants were transferred to SD conditions (8 hours of light and 16 hours of darkness, at 25/22°C, 40% relative humidity) until the flowering stage [64]. For rhythm assays, samples were collected every 4 hours over a period of 48 hours.

### Phenotypic statistics of flowering time

To assess the flowering time, the day of transplantation was designated as the first day, and the emergence of the first visible flower bud was recorded as the onset of flowering [65]. The budding stage was defined as the point at which the flower bud became visible, while full bloom stage refers to the point at which the florets of the inflorescence are fully expanded and show no further visible growth for each measurement, replicates were performed with 20 independent plants to ensure statistical reliability.

### Gene cloning and phylogenetic analysis

Total RNA was extracted from chrysanthemum “Jinba” using TRIzol (Invitrogen, Carlsbad, CA, USA) and treated with RNase-free DNase I (Thermo Scientific™, MA, USA), according to the manufacturer’s instructions. The first-strand cDNA was synthesized with SuperScript III reverse transcriptase (Invitrogen, Carlsbad, CA, USA). *CmTCP7* and *CmARF3* were isolated from “Jinba” using gene-specific primers 3-GSP1/2/3 and 5-GSP1/2/3 by performing 3′- and 5′-RACE. The full-length sequence of *CmTCP7* and *CmARF3* was verified with the primer pairs full-length-F/ -R. In this study, all primers were designed using Primer Premier 6.0 (San Francisco, USA) software and are listed in the Supplemental Table S3. The amplicons of *CmTCP7* and *CmARF3* were sequenced. The amino acid sequences of orthologues of CmTCP7 and CmARF3 in other species were obtained through BLAST searches (https://blast.ncbi.nlm.nih.gov/Blast.cgi), while those in *Arabidopsis* were obtained through the TAIR website (http://www.Arabidopsis.org/). Protein alignments were performed using DNAMAN 5.2.2 software (San Ramon, USA), and phylogenetic analysis was conducted utilizing MEGA 5.0 software with the neighbor-joining method, employing 1000 bootstrap replicates.

### Quantitative analysis of gene expression

Under LD conditions, apical buds and the third fully expanded leaves were collected when the plants had developed 14 leaves. After being transferred to SD conditions for three days, apical buds and the third fully expanded leaves of the plants were collected. For LD or SD treatment, three biological replicates were included, each biological replicate includes samples from three independent plants. The *Evo M-MLV* RT Kit with gDNA Clean for qPCR (Accurate Biotechnology) was used to synthesize cDNA from 1 μg of total RNA following the manufacturer’s instructions. A total of 30 ng of cDNA was used in 10 μl quantitative RT-PCR (qRT–PCR) reactions with Premix *Pro Taq* HS qPCR Kit (Accurate Biotechnology, HuNan, China) using Bio-Rad CFX (California, USA). *Elongation Factor 1α* (*CmEF1α*) was used as the reference gene. For quantification analysis of *CmTCP7* expression levels, the specific primers *CmTCP7-*Q-F and *CmTCP7-*Q-R located at ORF and 3′UTR, respectively, were designed. For quantification analysis of *CmARF3*, *CmCDM111L*, *CmFDL1,* and *CmFTL3* expression levels, the specific primers Q-F/R located in the ORF were designed. The specific primers for quantification analysis are listed in the Supplemental Table S3.

### Vector construction

For the BiFC assay, the ORFs of *CmTCP7*, *CmFDL1,* and *CmFTL3* genes without the termination codon were introduced into the pSATNA–nEYFP-N1 and pSATNA–cEYFP-N1 vectors. *CsFTL3* and *CsFDL1* are key genes involved in flowering in *C. seticuspe* under SD conditions [34]. *CmFTL3* is a key gene involved in flowering in *C. morifolium* [66], and the cloning primers for *CmFDL1* are listed in the Supplemental Table S3. pSATNA-nEYFP-N1–CmFDL1 and pSATNA-cEYFP-N1–CmFDL1 were generated using *EcoR* I/*Sal* I (NEB, 240 County Road Ipswich, MA, USA). pSATNA-cEYFP-N1–CmTCP7 were generated using *Xba* I/*Sal* I (NEB, 240 County Road Ipswich, MA, USA), and pSATNA-nEYFP-N1–CmFTL3 were generated using *Xba* I/*Xho* I. For the Y2H, the ORFs of *CmFDL1*, *CmFTL3,* and *CmTCP7* without the termination codon were constructed as pGBKT7–CmFDL1, pGADT7–CmFDL1, pGADT7–CmFTL3, and pGBKT7–CmTCP7, respectively, using the dual-enzyme digestion method. pGBKT7–CmTCP7 was constructed using *Nde* I and *Sal* I (NEB, 240 County Road Ipswich, MA, USA). pGADT7–CmFDL1 was generated using *BamH* I and *Xho* I (NEB, 240 County Road Ipswich, MA, USA), and pGBKT7–CmFDL1 was constructed using *BamH* I/*Sal* I (NEB, 240 County Road Ipswich, MA, USA). To obtain *anti-CmTCP7* transgenic plants, the reverse ORF of *CmTCP7* amplified with the primer pair CmTCP7-anti-pBIG-F/R was introduced into the pBIG vector by the dual-enzyme digestion method using *Sac* I and *Xba* I (NEB, 240 County Road Ipswich, MA, USA). To obtain *amiRCmARF3* transgenic plants, the plasmid pENTR™1A-amiRCmARF3 was constructed as previously described [67]. Furthermore, it was recombined with the binary vector pMDC32 by an “LR” reaction (Gateway™ LR Clonase™ II Enzyme Mix, Invitrogen, Carlsbad, CA, USA). To obtain the *OECmARF3* transgenic plants, the ORF of *CmARF3* was introduced into the pENTR™1A vector and was recombined with the binary vector pMDC32 by an “LR” reaction (Gateway™ LR Clonase™ II Enzyme Mix, Invitrogen, Carlsbad, CA, USA). The *CaMV 35S* promoter-driven *RLuc* and NOS terminator were inserted into the *EcoR* I site of the pUC19 vector by in-fusion cloning. The *CmTCP7* promoter-driven *luciferase* and NOS terminator were inserted into the *EcoR* I site of the pUC19_*RLuc* vector at the 5′end of the *RLuc* gene by in-fusion cloning. The 2× *CaMV 35S* promoter-driven *CmARF3* and NOS terminator were placed into the *Aat* II site of the pUC19_*RLuc*_*CmTCP7*pro:*luciferase* vector at the 5′ end of the *CmTCP7pro*:*Luc* by in-fusion cloning. The pUC19_*RLuc*_*CmTCP7pro*:*luciferase* vector construct was used as a negative control. The *CmCDM111L* promoter-driven luciferase gene along with the NOS terminator was inserted into the *EcoR* I site of pUC19_RLuc, positioned at the 5′ end of the *RLuc* gene. Finally, the 2× *CaMV 35S* promoter-driven *CmARF3* gene along with the NOS terminator was inserted into the *Aat* II site of the pUC19_*RLuc*_*CmCDM111Lpro*:*luciferase* vector. All primers are listed in the Supplemental Table S3.

### Chrysanthemum transformation

Chrysanthemum transformations were performed with Agrobacterium-mediated leaf disk infection [68]. Briefly, leaves from the upper portion of 1-month-old tissue culture plantlets were selected and cut into approximately 5 × 5 mm leaf disks, ensuring that the edges were wounded. After callus formation at the wound sites, the disks were infected with *Agrobacterium* for 8 minutes, followed by selection and regeneration on MS medium containing antibiotics. The pBIG–CmTCP7 vector was used to generate *anti-CmTCP7* transgenic plants. The pMDC32–CmARF3 vector was used to generate *OECmARF3* transgenic plants, and the pMDC32–amiRCmARF3 vector was used for the preparation of *amiRCmARF3* transgenic plants. The *OECmARF3* and *amiRCmARF3* transgenic plants were selected on MS medium containing 8 mg/L hygromycin, and the *anti-CmTCP7* transgenic plants were selected on MS medium containing 7.5 mg/L kanamycin. DNA and RNA were extracted from the putative transgenic and WT plants, respectively. For the primary screening of putative transgenic plants, DNA and vector primers were used for PCR amplification. The relative expression levels of the positive transgenic plants were determined using the above-mentioned primers through qRT–PCR analysis.

### Promoter cloning

DNA was extracted from chrysanthemum “Jinba” using the CTAB DNA extraction protocol. The promoters of *CmTCP7* were cloned using the TaKaRa LA PCR™ *in vitro* Cloning Kit (TaKaRa, Kyoto, Japan) and verified by high-fidelity PCR using CmTCP7pro-F/R primers (Supplemental Table S3). The 1657 bp region upstream of the *CmCDM111L* ORF was cloned and verified by high-fidelity PCR using CmCDM111Lpro-F/R primers (Supplemental Table S3). The promoters were constructed into the pGEM^®^-T Easy Vector using TA cloning (Promega, Wisconsin, USA).

### A yeast one-hybrid system

The upstream regulatory genes of *CmTCP7* were identified using the AuxRE (3X [5′-TGTCTC-3′]) in the yeast one-hybrid cDNA library of chrysanthemum (constructed by Invitrogen, Carlsbad, CA, USA), and verification experiments were performed using a Clontech system (Clontech, Mountain View, CA, USA). The whole *CmTCP7* promoter was cloned into the pHis vector using the dual-enzyme digestion method with *EcoR* I and *Xba* I (NEB, 240 County Road Ipswich, MA, USA) restriction enzyme cutting sites. The plasmids of pGADT7, pGADT7–CmARF3, and pHis-CmTCP7pro were transferred into yeast strain *Y1H* (Clontech, Mountain View, CA, USA), and the pGADT7 vector was used as a negative control. The growth of the yeast cells transformed with the gene of interest was assessed on SD/-His-Ura-Leu medium supplemented with 30 mM 3-aminotrizole (3-AT).

### EMSA

*CmARF3* with an N-terminal GST fusion tag in the pET-60-DEST vector was generated. In the EMSA experiments, synthetic double-stranded DNA oligonucleotides corresponding to the *CmTCP7* promoter sequence were generated, encompassing the binding sites for AuxRE. The oligonucleotide probes were 5′ end-labeled with biotin. CmARF3 protein was expressed in *BL21* cell cultures incubated overnight at 140 rpm/min with 0.1 mM isopropyl-β-D-thiogalactoside (IPTG) and isolated using GST beads (Beaver Beads™ GSH, Suzhou, China). In the binding reactions, 6–10 μg of the CmARF3 protein was incubated with probes using the Light Shift™ Chemiluminescent EMSA Kit (Thermo Scientific™, MA, USA). Cold probes were made up of unlabeled probes that share the same sequence as biotin probes.

### Plant ChIP qPCR assay

The CmARF3 polyclonal antibody was induced in mice using full-length CmARF3 protein (pET-60-DEST–CmARF3). The CmARF3 polyclonal antibody was prepared by the Institute of Genetics and Developmental Biology (Beijing, China), and the antiserum was purified using a Protein A Sepharose column. Expanded leaves of chrysanthemum plants were sampled. The plant tissues were fixed in formaldehyde (final concentration, 1%), under vacuum, until the tissues sank below the liquid surface. Fixed tissues were homogenized, and the chromatin complexes were isolated and fragmented by sonication using a sonifier (Diagenode Bioruptor Sonication Device Bioruptor Pico, NJ, USA). For immunoprecipitation, the solubilized chromatin and 20 μl CmARF3 antibody were incubated at 4°C with mixing overnight. Following this procedure, the resulting antibody complex was then isolated using protein A beads (Thermo Scientific™, MA, USA). After washing and elution, the eluents were incubated at 65°C overnight to reverse cross-link. Protein digestion and purification of the eluents were then performed the following day. The purified DNA was used for real-time quantitative PCR (RT-qPCR) analyses. Primers used for ChIP-qPCR are listed in Supplemental Table S3.

### Transient transformation dual-luciferase assays in chrysanthemum protoplasts

The protoplast transformation assay was performed as previously described [33]. When chrysanthemum “Jinba” cuttings reached eight leaves on the rooting medium, protoplasts were isolated from the upper leaves. The leaves were immersed in a 0.4 M mannitol solution and then cut into approximately 1.0-mm long strips. Following this, the cut leaves were submerged in a 20 ml enzyme solution composed of 0.4 M mannitol, 20 mM KCl, 20 mM Mes-KOH (pH 5.7), 10 mM CaCl_2_, 1.5% (wt/vol) Cellulase R10, and 0.4% (wt/vol) Macerozyme R10. The samples were shielded from light and incubated for 4 hours at 24°C. The protoplasts were subsequently filtered through Miracloth. To stop the reaction, an equal volume of W5 solution containing 154 mM NaCl, 125 mM CaCl_2_, 5 mM KCl, 2 mM Mes-KOH (pH 5.7), and 5 mM glucose was added. The protoplasts were washed twice with W5 solution and then resuspended in MMG solution containing 0.4 M Mannitol, 15 mM MgCl_2_, and 4 mM Mes-KOH (pH 5.7). After mixing plasmids of different combinations (each plasmid 10 μg), they were added to 200 μl of protoplasts, followed by the addition of 220 μl of 40% PEG solution, and gently mixed. After a 20-minute incubation in darkness, 800 μl of W5 solution was added to terminate the reaction. After centrifugation to remove the supernatant, 1 ml of W5 solution was added, and the solution was incubated at 24°C for 20 hours.

### BiFC assay

For the BiFC assay, pSATNA-nEYFP-N1–CmFTL3, pSATNA-cEYFP-N1–CmTCP7, pSATNA-cEYFP-N1–CmFDL1, and pSATNA-nEYFP-N1–CmFDL1 vectors were constructed and introduced into Agrobacterium, including the nuclear localization sequence (NLS) vector, which had an m-Cherry tag. Each of the following interaction pairs in *Agrobacterium* cells was infiltrated into *N. benthamiana* leaves: pSATNA-nEYFP-N1–CmFTL3/pSATNA-cEYFP-N1–CmTCP7, pSATNA-nEYFP-N1–CmFDL1/pSATNA-cEYFP-N1–CmTCP7, and pSATNA-nEYFP-N1–CmFTL3/pSATNA-cEYFP-N1–CmFDL1. Simultaneously, negative controls were conducted using empty vectors in conjunction with each fusion construct. The m-Cherry fluorescent marker with a nuclear localization signal was included in each combination and acted as a positive control. The plants were incubated in the dark for 16 h at 22°C and grown for 2 days under LD conditions before observation. The fluorescent signals of GFP, YFP, and mCherry-NLS and bright-field images were captured using the Leica TCS SP8 (Wetzlar, Germany).

### Co-IP assay and immunoblot analysis

The ORFs of CmFDL1 and CmFTL3 with the termination codon were introduced into pEarleyGate 201(HA), and *CmTCP7* was cloned into pEarleyGate 202 (Flag) vector. The plasmids were transiently introduced into tobacco *N. benthamiana*. Before harvesting samples, the plants were grown under LD conditions for three days. Leaves were collected, and protein extraction was performed using the following extraction solution: 50 mM Tris–HCl, pH 7.4; 150 mM NaCl; 1 mM EDTA; 10% Glycerol; 1% TritonX-100; Cocktail (Roche, Basel, Switzerland). Immunoprecipitations were performed using the anti-HA affinity matrix, and immunoaffinity purification of tagged proteins was performed with the HA peptide (Roche, Basel, Switzerland, Cat. No. 11815016001; HA peptide, MedChemexpress, NJ, USA, CAS No.: 92000-76-5).

Co-IP western blots were performed using anti-HA high affinity (1:5000; ROCHE, Basel, Switzerland, 3F10) and antiflag antibody (1:2000; SIGMA, Darmstadt, Germany, F1804), followed by a secondary antibody (1:5000, anti-rat IgG HRP-conjugated, abcam, Cambridge, England; 1:10000, antimouse IgG, HRP-conjugated, abcam, Cambridge, England). Samples were fractionated on 7.5% Mini-PROTEAN TGX Gels (Bio-Rad, California, USA) and blotted onto polyvinylidene fluoride membranes using a semidry electrophoresis apparatus (Bio-Rad, California, USA). Chemiluminescence was detected with the Clarity Western ECL substrate (Bio-Rad, California, USA).

### Luciferase complementation assay

The full-length coding sequence of CmFTL3 and CmFDL1 was ligated into pCAMBIA-nluc and pCAMBIA-cluc vectors, respectively, to generate CmFTL3-nLUC and CmFDL1-cLUC. The Cluc-/Nluc-derivative constructs and CmTCP7-HA were transformed into *Agrobacterium tumefaciens* strain *GV3101* and cultured for two days. After cultivation, the *Agrobacterium* strains were resuspended and mixed with a ratio of 1:1:3 in the CmFTL3–CmFDL1cLUC–CmTCP7nLUC combination, while the other four combinations (CmFTL3nLUC–CmFDL1cLUC, CmFTL3nLUC–pCAMBIAcLUC, CmFDL1cLUC–pCAMBIAnLUC, CmTCP7nLUC–pCAMBIAcLUC, and CmTCP7cLUC–pCAMBIAnLUC) were mixed in a ratio of 1:1. These mixtures were then injected into *N. benthamiana* leaves. The plants were incubated in the dark for 16 h at 22°C and grown for 2 days under LD conditions before observation. Luciferase activity was detected with D-luciferin (Biovision, MA, USA) and the GloMax® 20–20 Luminometer (Promega, Wisconsin, USA).

### Virus-induced gene silencing

*CmTCP7* was silenced in *amiRCmARF3* transgenic chrysanthemum plants using a virus-based miRNA expression system, as we described before [64]. The primers used in construction are listed in the Supplemental Table S3. The RS300 plasmid was employed to clone *amiRCmTCP7*, which was subsequently transferred into the CaLCuV vector and introduced into *A. tumefaciens* strain *GV3101*. Prior to infiltration, a 1:1 (v/v) mixture of pCVB and either CaLCuV (control) or CaLCuV–CmTCP7 was incubated in the dark at 28°C for 4 hours. Cuttings from *amiRCmARF3–22* plants were excised and vacuumed in infiltration buffer at 0.7 MPa for 10 min. Cuttings were placed in the dark at 10°C for 1–3 days, then planted into pots containing a 1:3 (v/v) mixture of garden soil and vermiculite. These plants were grown under LD conditions for 3 weeks, and then transferred to SD. A newly emerged third leaf from the apex of the plant was sampled for expression-level analysis of *CmTCP7*. Two independent lines of *amiRCmARF3–22-CaLCuV* and *amiRCmARF3–22/CaLCuV–CmTCP7* lines were used for flowering time observation.

### Hormone treatments

IAA (Sigma-Aldrich, 87–51-4) and PEO–IAA (2-(1H-indol-3-yl)-4-oxo-4-phenylbutanoic acid, Sigma-Aldrich, 6266-66-6) were dissolved in DMSO to create stock solutions. The stock solutions were diluted with distilled water to the following concentrations: IAA at 500 μM and 1000 μM, and PEO–IAA at 100 μM. A control treatment was performed using DMSO at the same concentration as in the experimental treatments. For the treatment of chrysanthemum “Jinba,” plants at 14 fully expanded leaf stage were selected, plants at this stage have completed their vegetative growth phase and are physiologically prepared for the transition to reproductive growth when subjected to SD conditions. The plants were treated with IAA and PEO–IAA solutions by foliar spray just before the onset of night to minimize the degradation of auxin. The spray was applied until droplets dripped from the leaves. Treatments were administered every three days, and after three times treatments, the plants were transferred to SD conditions. IAA and PEO–IAA were then applied every three days until the plants began to flowering. For each treatment, 15 individual plants were included.

### Statistical analysis

Data were analyzed using SPSS v17.0 software (SPSS Inc., Chicago, IL, USA). Results are presented as the mean ± standard deviation. For comparisons between two groups, statistical significance was determined using Student’s *t*-test. Specifically, a *P*-value greater than 0.01 but less than or equal to 0.05 was indicated with one asterisk (*), a *P*-value less than or equal to 0.01 was indicated with two asterisks (**), and a *P*-value greater than 0.05 was considered not statistically significant. All experiments were performed with biological replicates (*n* = [number of biological replicates]).

#### Accession numbers

Genes referenced in this article are available at GenBank with the accession numbers: MZ032006 (*CmARF3* ORF); MZ032007 (*CmTCP7* ORF); MZ032008 (*CmTCP7* promoter).

## Supplementary Material

Web_Material_uhaf095

## Data Availability

The data underlying this article are available in the article and in its online supplementary material.
