# Supplementary material for: CmARF3–CmTCP7 module regulates flowering time in chrysanthemum (Chrysanthemum morifolium)
Source: Hortic Res. 2025 May 23;12(7):uhaf095. doi: 10.1093/hr/uhaf095 (PMC12099206; doi:10.1093/hr/uhaf095)
Supplement: Web_Material_uhaf095 [file web_material_uhaf095.zip › Supplemental_Figures_uhaf095.docx]

**Supplemental Figures**

**
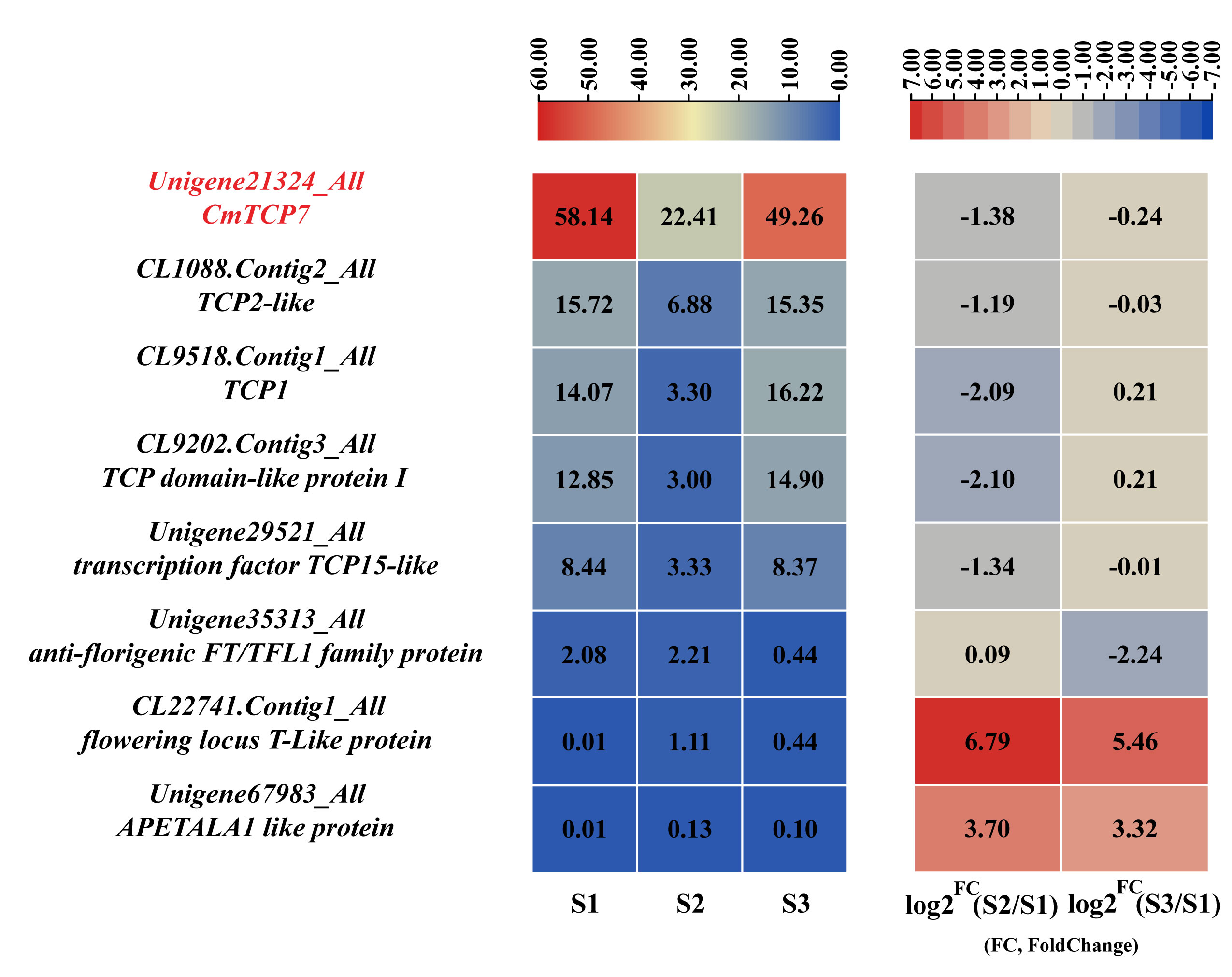
**

**Figure S1.** The differentially expressed genes of TCP family members and flowering-related genes in transcriptome analysis. S1, the apex of plants before flower bud differentiation (long-day conditions). S2, the apex of plants under the doming stage (3 days after transitioning to short-day conditions). S3, the involucre differentiation process stage (7 days after transitioning to short-day conditions). Sampling selected under a stereo microscope. *CmTCP7* is highlighted in red.


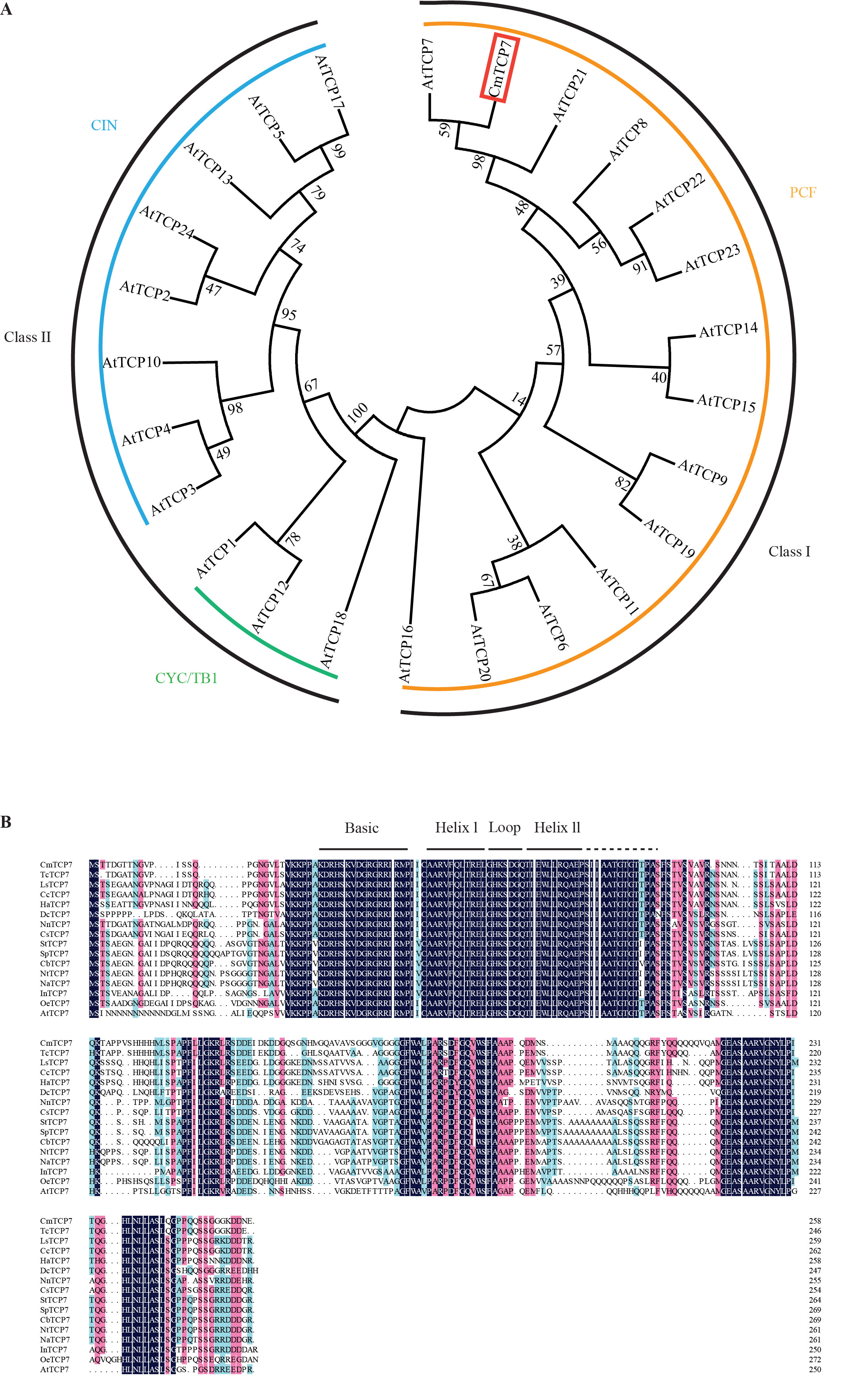


**Figure S2.** Neighbor-joining phylogenetic tree analyses and sequence alignments of *CmTCP7* in *Chrysanthemum morifolium* ‘Jinba’. (A) Phylogenetic analysis of *AtTCP* genes and *CmTCP7* gene (boxed) were performed with neighbor-joining tree constructed by MEGA 5.0 software. Bootstrap values >50% for 1,000 bootstrap replications are shown on each branch. orthologs of *AtTCP* genes were used for the analyses and formed distinct clades (Class I group and Class II group). (B) Protein alignments of TCP7s BHLH domain from *Chrysanthemum morifolium* ‘Jinba’(*CmTCP7*); *Arabidopsis thaliana* (AtTCP7, OAO91023.1), *Tanacetum cinerariifolium* (TcTCP7, GEV09021.1); *Lactuca sativa* (LsTCP7, XP_023736444.1); *Cynara cardunculus* var. scolymus (CcTCP7, XP_024994214.1); *Helianthus annuus* L. (HaTCP7, XP_022005986.1); *Daucus carota subsp. sativus* (DcTCP7, XP_017238778.1); *Solanum tuberosum* (StTCP7, XP_015169841.1); *Solanum pennellii* (SpTCP7, XP_015073177.1); *Nelumbo nucifera* (NnTCP7, XP_010249637.1); *Olea europaea* var. sylvestris (OeTCP7, XP_022868503.1); *Camellia sinensis* (CsTCP7, XP_028051557.1); *Ipomoea nil* (InTCP7, XP_019163704.1); *Capsicum baccatum* (CbTCP7, PHT54139.1); *Nicotiana attenuata* (NaTCP7, XP_019231460.1); *Nicotiana tabacum* (NtTCP7, XP_016444705.1). The single underline indicates the conserved bHLH domain. The black background represents 100% identity, the red background represents 75% identity and the blue represents 50% identity.


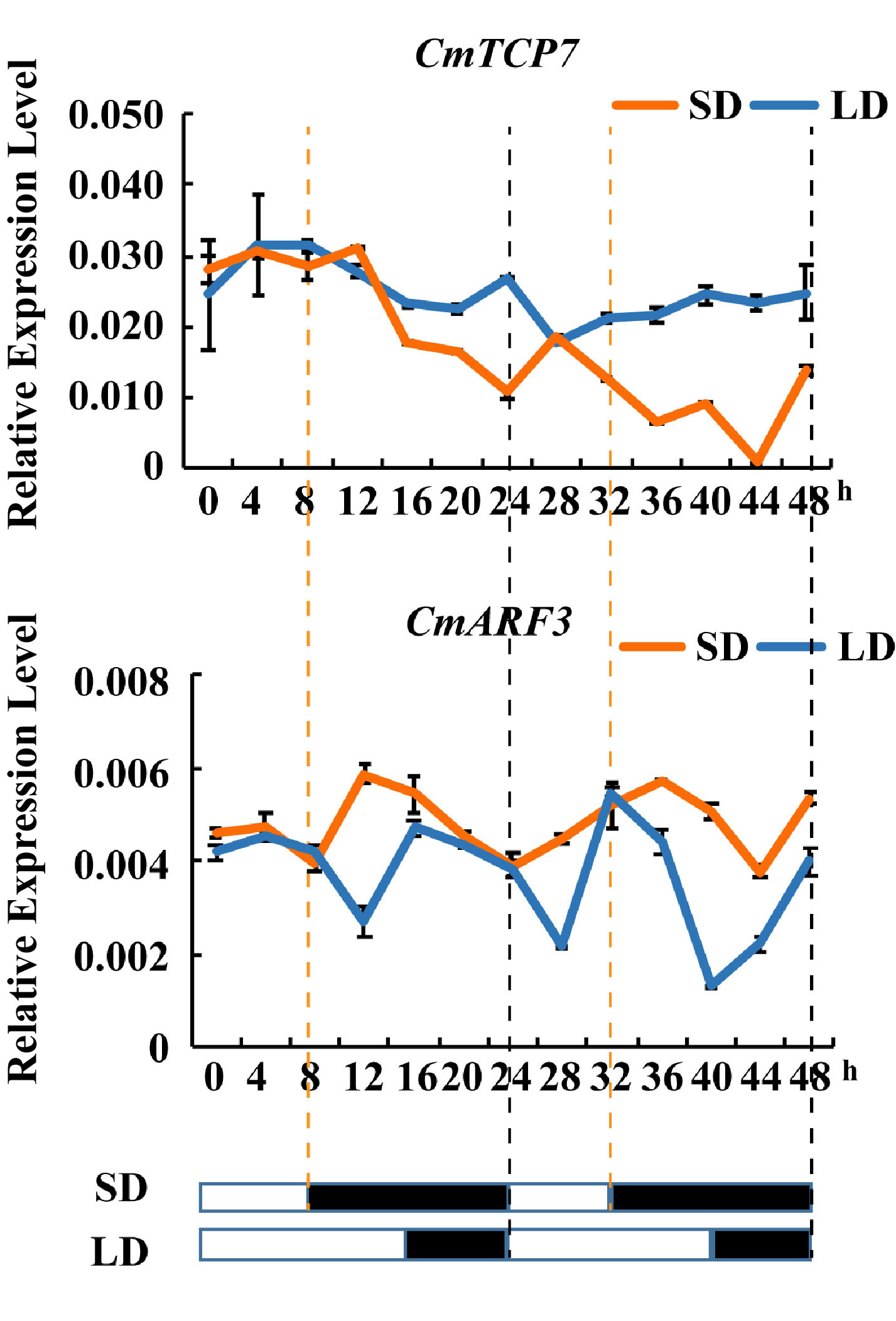


**Figure S3.** The diurnal expression of *CmTCP7* and *CmARF3* over 48-h under short-day (SD) or long-day (LD) conditions. The expression levels of *CmTCP7* and *CmARF3* in WT leaves under the different conditions. The values are presented as the mean ± standard deviation (n = 3).


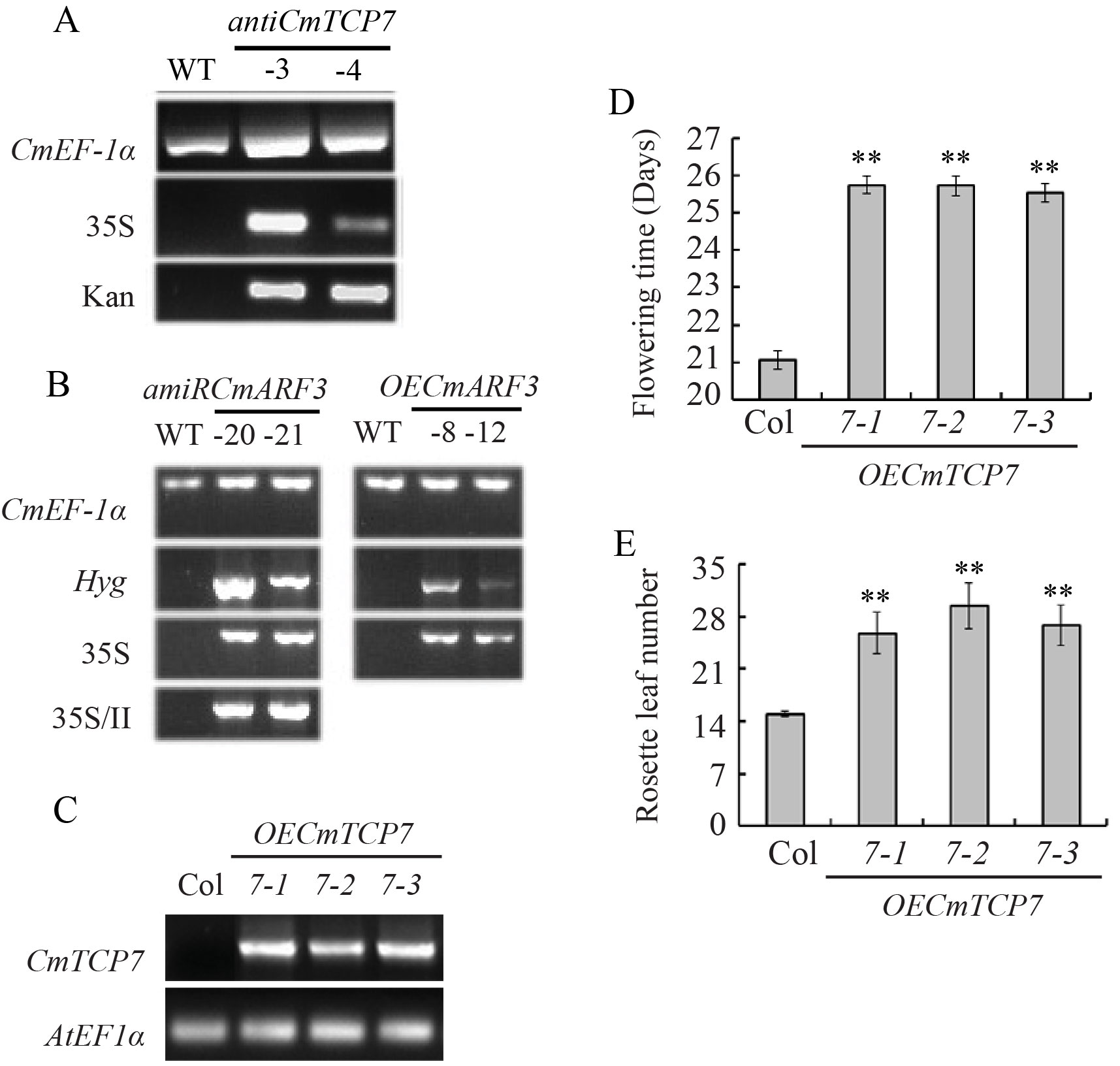


**Figure S4.** Identification of transgenic chrysanthemum lines and ectopic expression of *CmTCP7* in Col background Arabidopsis (Col-0). (A) PCR verification of the positive *antiCmTCP7* transgenic lines. *CmEF1α* was used as the reference gene. 35S and Kan are fragments specifically amplified from the backbone of the vector. (B) Identification of the positive *amiRCmARF3* and *OECmARF3* chrysanthemum ‘Jinba’ transgenic lines at the DNA level. (C) RT-PCR analyses of T3 overexpression *CmTCP7* transgenic *Arabidopsis* lines. *AtEF1α* is the reference gene. (D) The flowering time of wild-type and T3 transgenic lines, Col-0 is wild-type, *7-1*, *7-2*, and *7-3* are transgenic overexpression plants of *CmTCP7*. The values are presented as the mean ± standard deviation (**, *p* < 0.01; Student’s *t*-test; n = 10) (E) The number of rosette leaves of wild-type and T3 transgenic lines. The values are presented as the mean ± standard deviation (**, *p* < 0.01; Student’s *t*-test; n = 10)


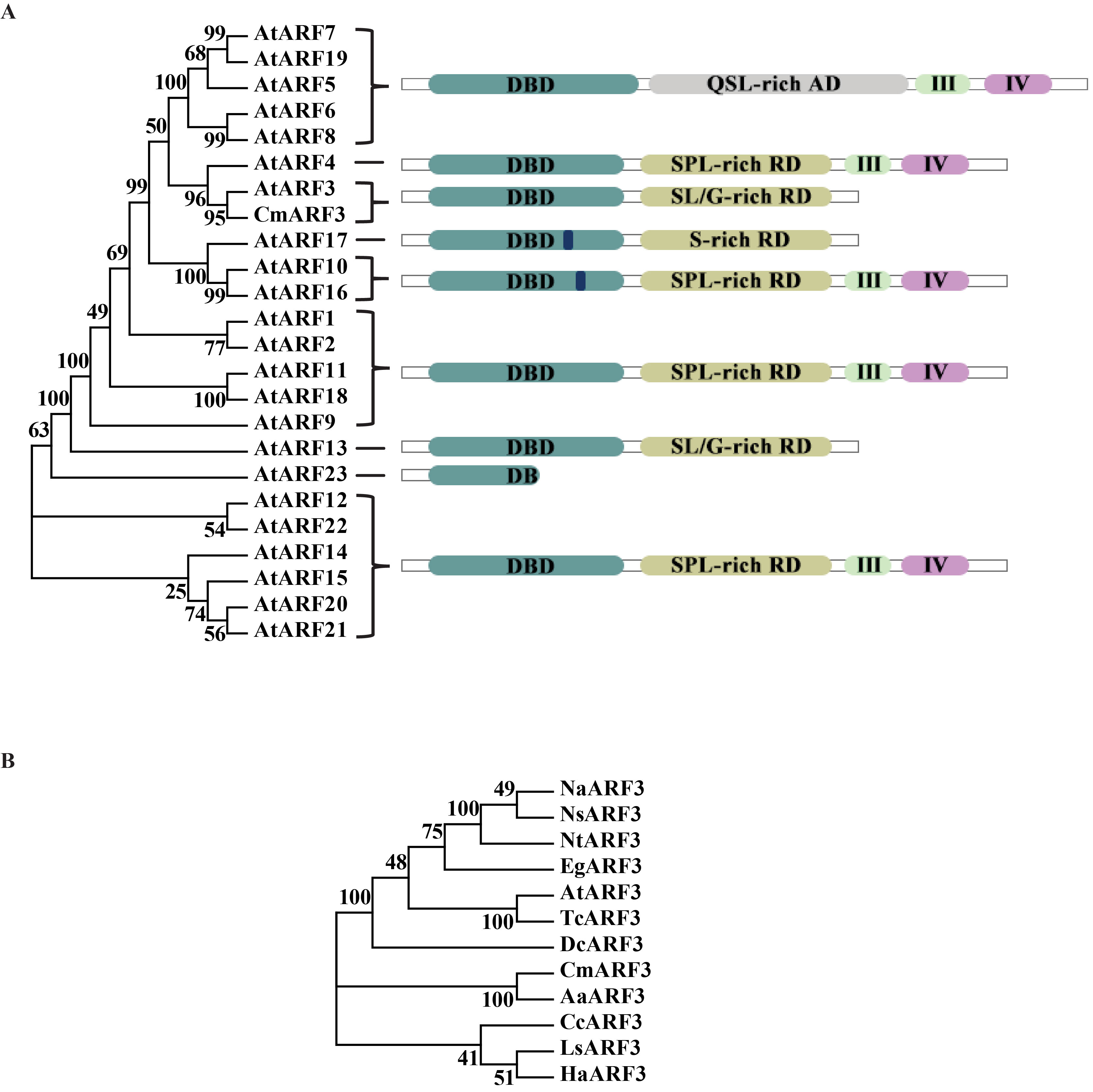


**Figure S5.** Sequence alignment and neighbor-joining phylogenetic tree analyses show that *CmARF3* is conserved in chrysanthemum.

(A) Phylogenetic analysis of *AtARF* genes and *CmARF3* gene were performed with the Neighbor-Joining tree, which is constructed using MEGA 5.0 software. Bootstrap values >50% for 1,000 bootstrap replications are shown on each branch. The right sections showed structural analyses of ARF proteins in Arabidopsis and chrysanthemum. (B) Protein alignments of ARFs: *Theobroma cacao* (TcARF3, EOY28143.1); *Lactuca sativa* (LsARF3, XP_023746845.1); *Cynara cardunculus* var. scolymus (CcARF3, XP_024989421.1); *Helianthus annuus* L. (HaARF3, XP_021975677.1); *Daucus carota subsp. sativus* (DcARF3, XP_017229595.1); *Nicotiana attenuata* (NaARF3, XP_019226082.1); *Nicotiana tomentosiformis* (NtARF3, XP_018623263.1); *Nicotiana sylvestris* (NsARF3, XP_009799485.1); *Erythranthe guttata* (EgARF3, XP_012849069.1); *Arabidopsis thaliana* (AtARF3, AT2G33860.1); *Artemisia annua* L. (AaARF3, PWA88762.1).


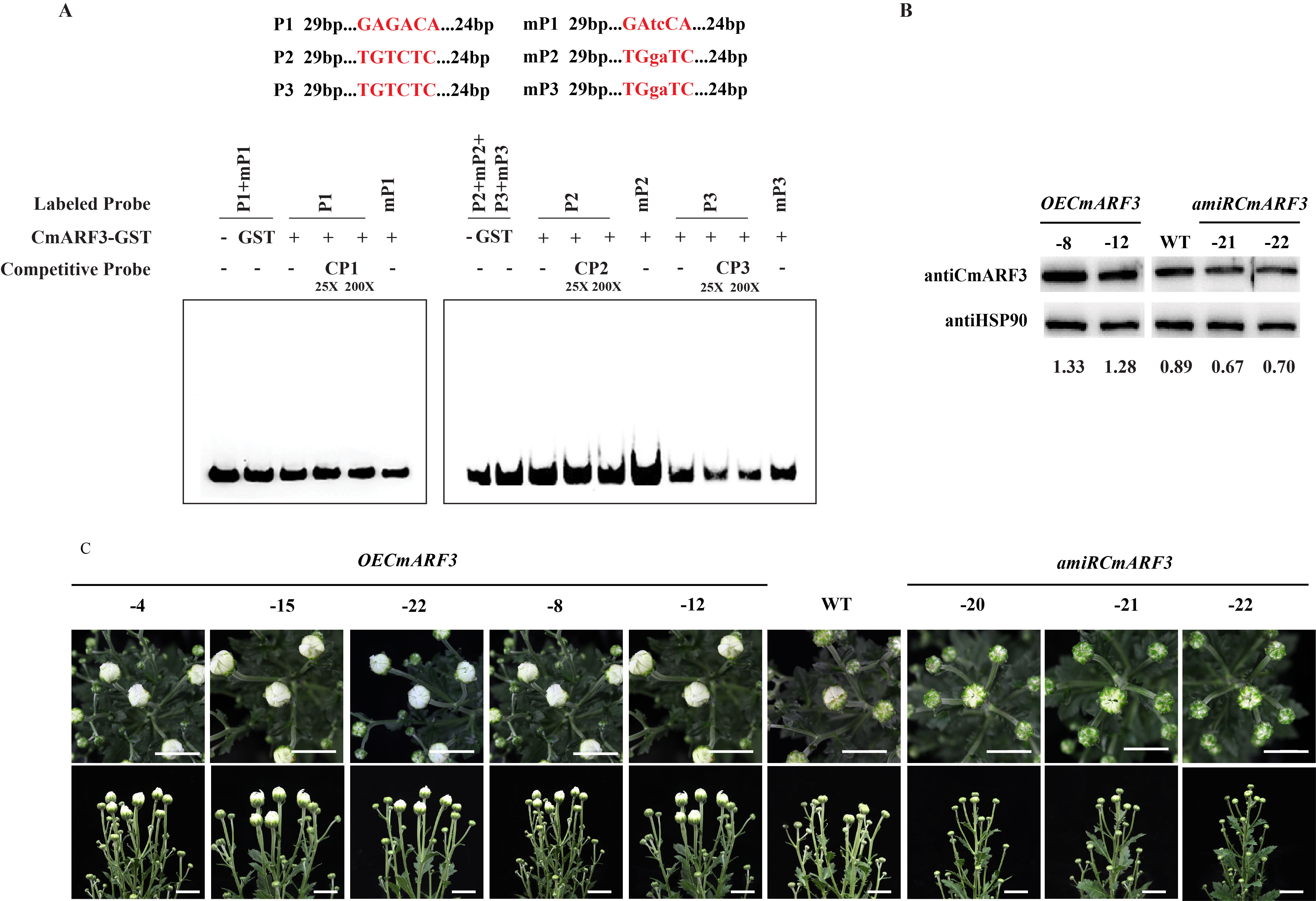


**Figure S6.** Electrophoretic mobility shift assay (EMSA) analyses and *CmARF3* transgenic chrysanthemum plant phenotypes. (A) EMSA analyses of the binding of CmARF3 to the AuxRE elements in the *CmTCP7* promoter. mP1/2/3 represent the mutant probes, in which the ce1, ce2 and ce3 were mutated. The TGTCTC AuxRE was mutated to TGgaTC and the reverse GAGACA was mutated to GAtcCA. No binding of recombinant CmARF3 proteins to the P1, P2, P3 probe or to the mP1, mP2, mP3 mutant probes was found. The unlabeled probe CP1, CP2 and CP3 were used as cold probes. The untreated GST protein isolates were used as the control. GST, GST-CmARF3, biotin-probe, labeled mutated probe, and unlabeled probe at a 20× and 100× molar excess were present (+) or absent (-) in each reaction. (B) Upper panel, western blotting result showing the different accumulation levels of CmARF3 protein in WT and *CmARF3* transgenic plants. HSP90 serves as a protein loading control. (C) Phenotypes of WT, *OECmARF3* and *amiRCmARF3* transgenic plants. Scale Bars, 2 cm.


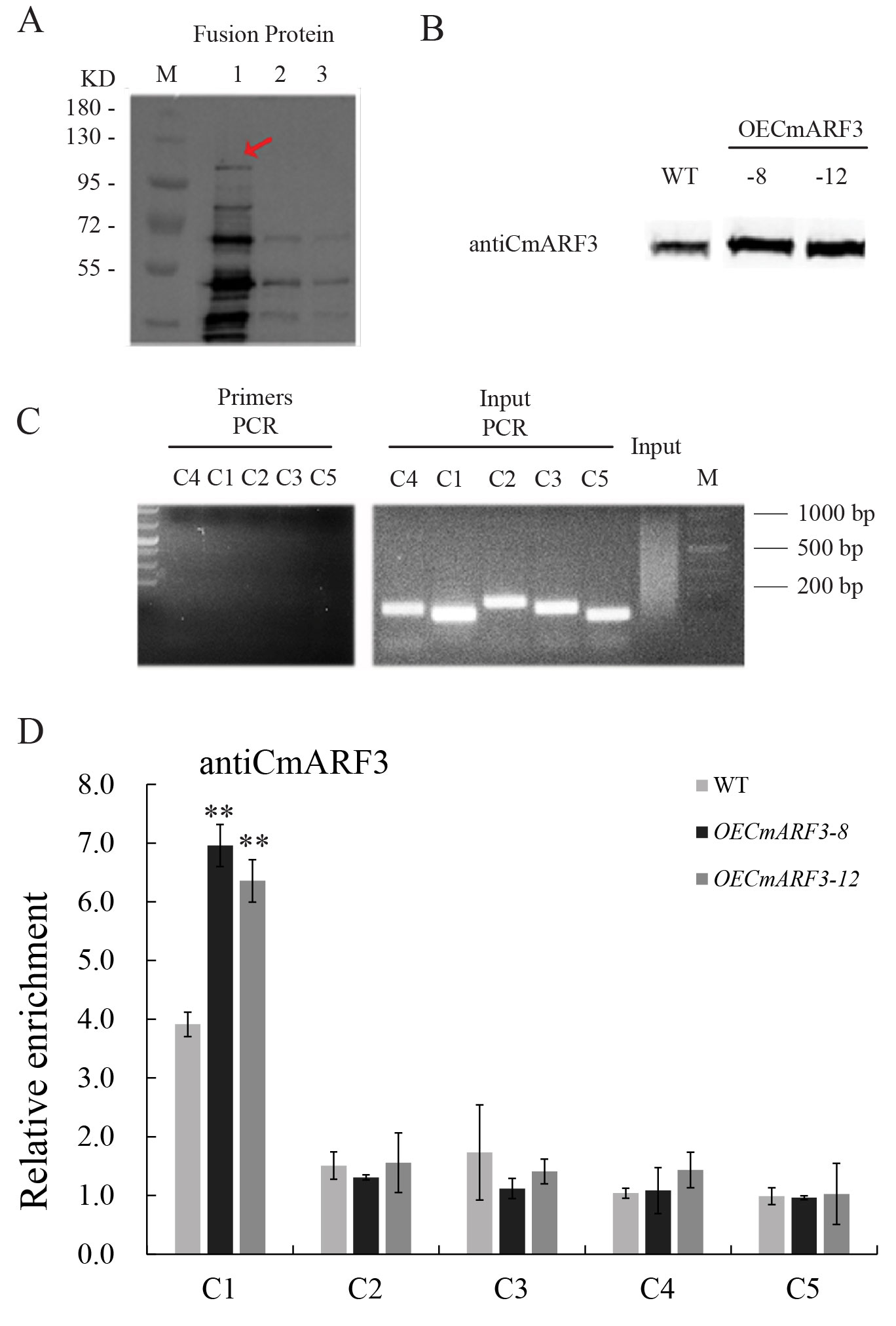


**Figure S7.** Identification of CmARF3 antibody and analysis of its immunity efficiency. (A) Immunodetection of CmARF3 antibody using CmARF3-GST fusion protein expressed in prokaryotic cells. The target fragment of the CmARF3-GST fusion protein is indicated by the red arrow. Lanes 1, 2, and 3 show CmARF3-GST fusion proteins diluted at 100×, 1000×, and 5000×, respectively. The anti-CmARF3-specific polyclonal antibody was diluted 1000× for this assay. M, Marker. (B) Immunodetection of CmARF3 antibody and plant endogenous target protein. The anti-CmARF3-specific polyclonal antibody was diluted 1000× in this assay. (C) Specific identification and size determination of chromatin immunoprecipitation (ChIP) fragments. M, Marker. (D) Relative binding of CmARF3 to the promoter of *CmTCP7*. The values are presented as the mean ± standard deviation (**, p < 0.01; Student’s *t-*test; n = 3).


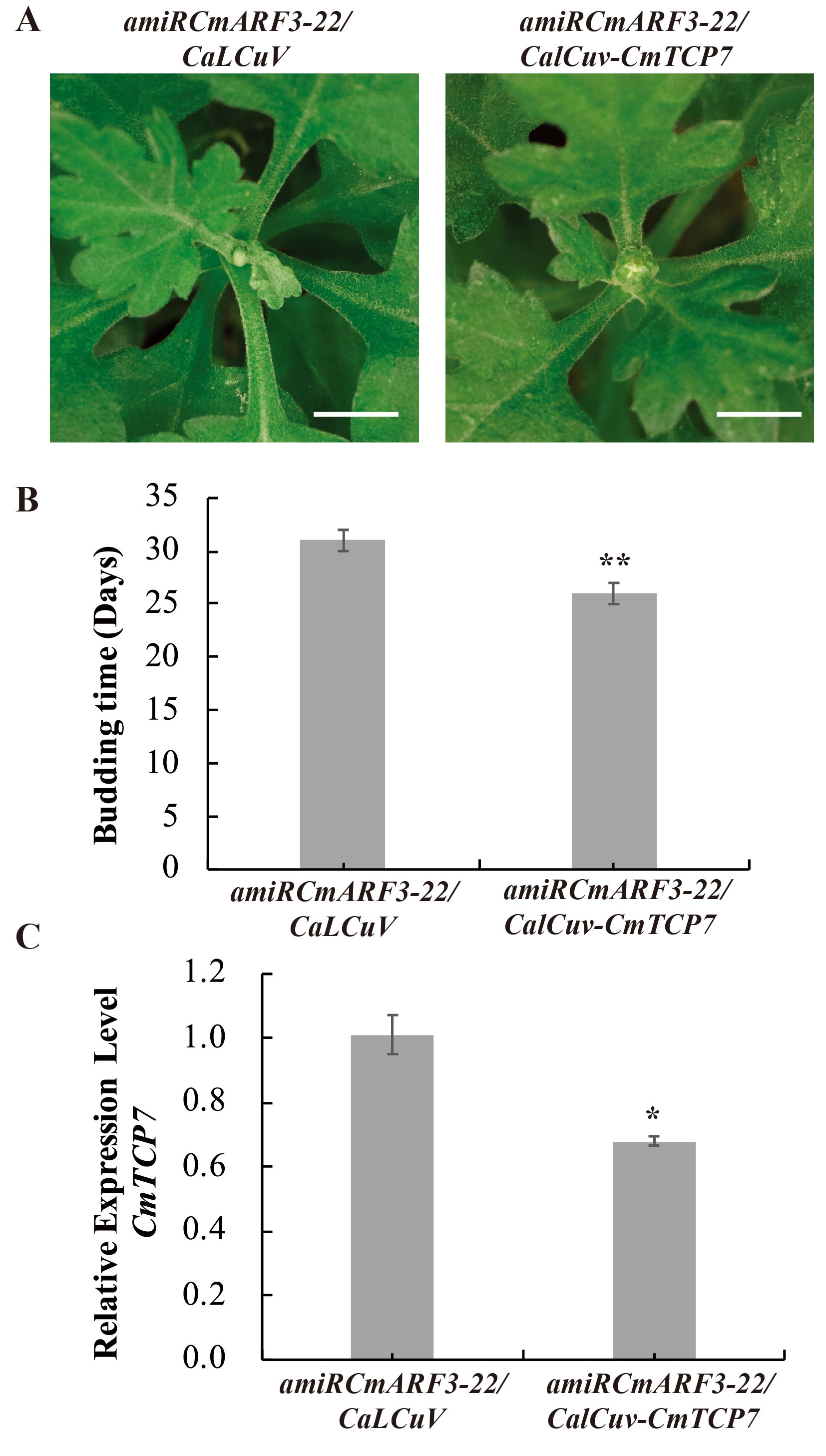


**Figure S8.** Genetic relationship analysis between *CmTCP7* and *CmARF3*. (A) and (B) Phenotypes and budding time of virus-induced *CmTCP7* gene silencing in *amiRCmARF3* transgenic plants. The values are presented as the mean ± standard deviation (**, p < 0.01; Student’s *t-*test; n = 3). Scale Bars, 1 cm. (C) Relative expression level of *CmTCP7* of virus-induced *CmTCP7* gene silencing in *amiRCmARF3* transgenic plants compared to control (CaLCuV empty vector). CaLCuV empty vector infestation in *amiRCmARF3* transgenic plants were used as a control. The values are presented as the mean ± standard deviation (*, p < 0.05; Student’s *t-*test; n = 3).


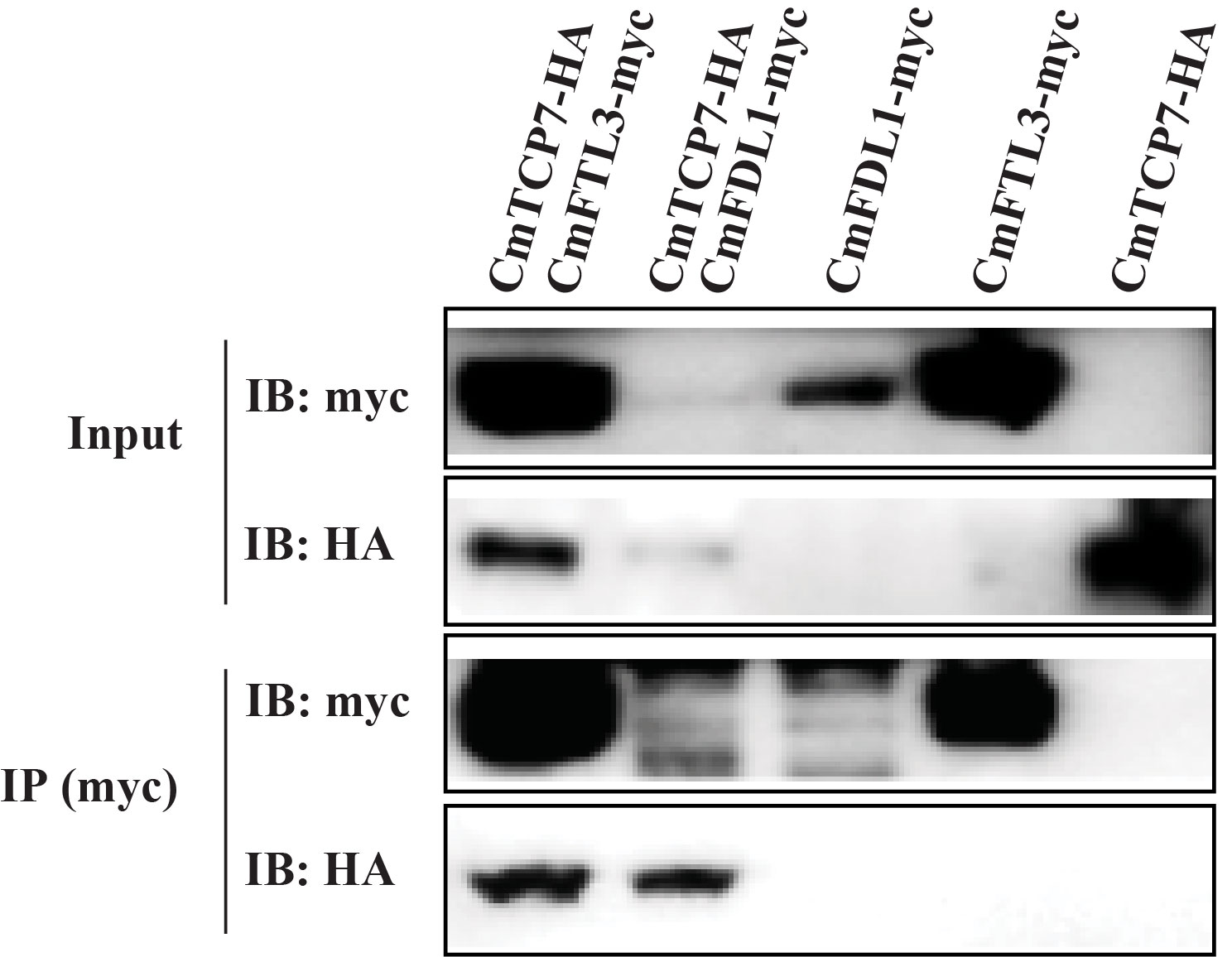


**Figure S9.** Co-IP assays of CmTCP7, CmFDL1 and CmFTL3 interactions in chrysanthemum protoplasts. myc-CmFDL1, myc-CmFTL3 and HA-TCP7 immunoprecipitated complexes were detected in Co-IP analysis input. myc-CmFDL1 and myc-CmFTL3 were immunoprecipitated using anti-myc antibody and co-immunoprecipitated HA-TCP7 was then detected using anti-HA antibody.


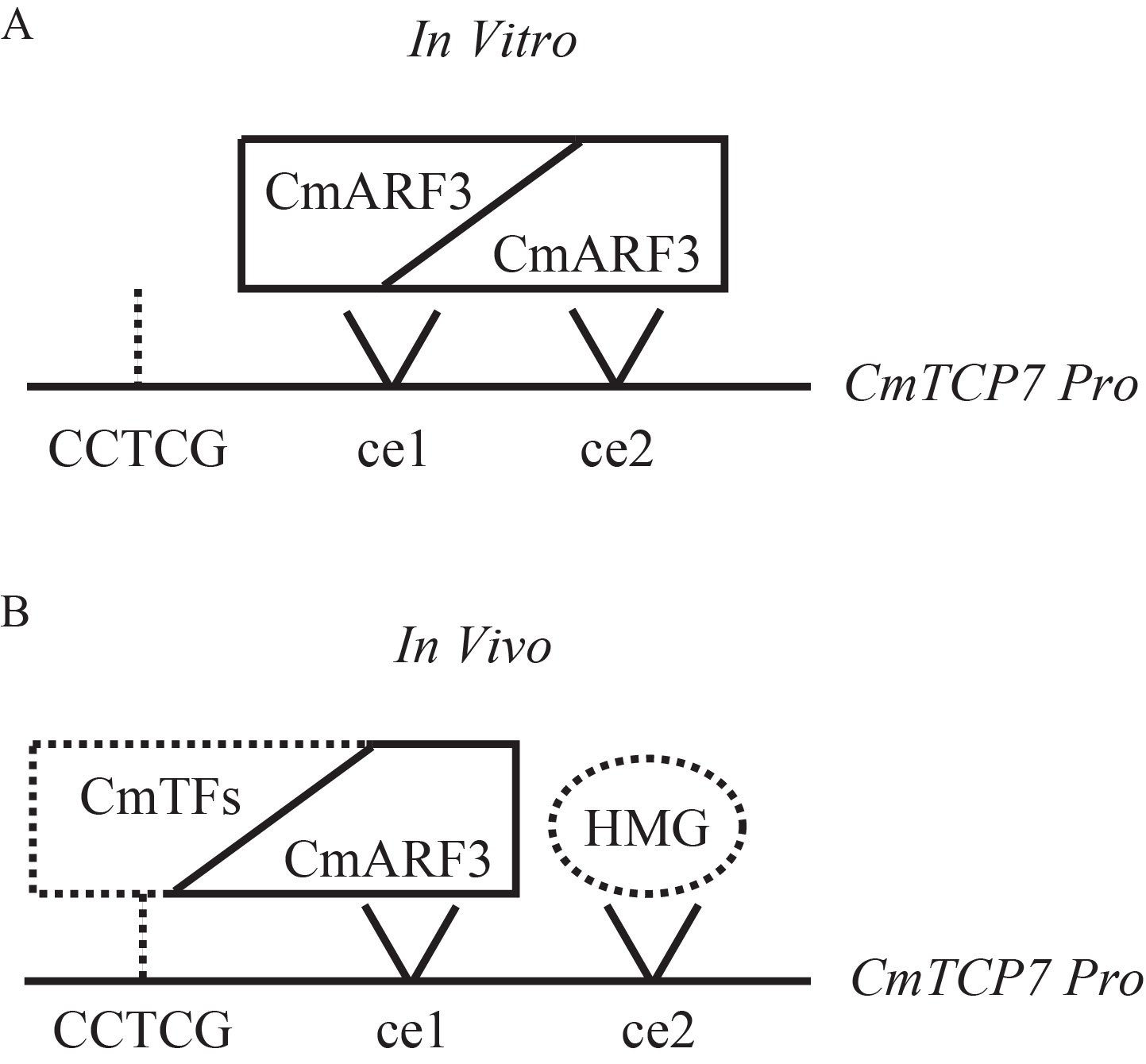


**Figure S10.** Hypothetical model of CmARF3 binding the *CmTCP7* promoter *in vitro* and *in vivo*. (A) CmARF3 probably required to form as a homodimer on palindromic TGTCTC AuxREs for stable binding *in vitro*. Sequence analysis found a candidate constitutive element CCTCG (the D1 component of GH3) upstream of the ce1. However, under *in vitro* conditions, the CCTCG motif lacks additional transcription factors for binding. (B) *In vivo*, CmARF3 is capable of interacting with process-specific transcription factors, where the dimerization state of the complex determines the selection of downstream targets. These transcription factors may also potentially bind to the CCTCG motif. In addition, some proteins like a large number of high mobility group (HMG)-like proteins may competitively bind to a TGTCTC site with CmARF3, which presumably resulted the un-enrichment of ce2 AuxRE in ChIP-qPCR.


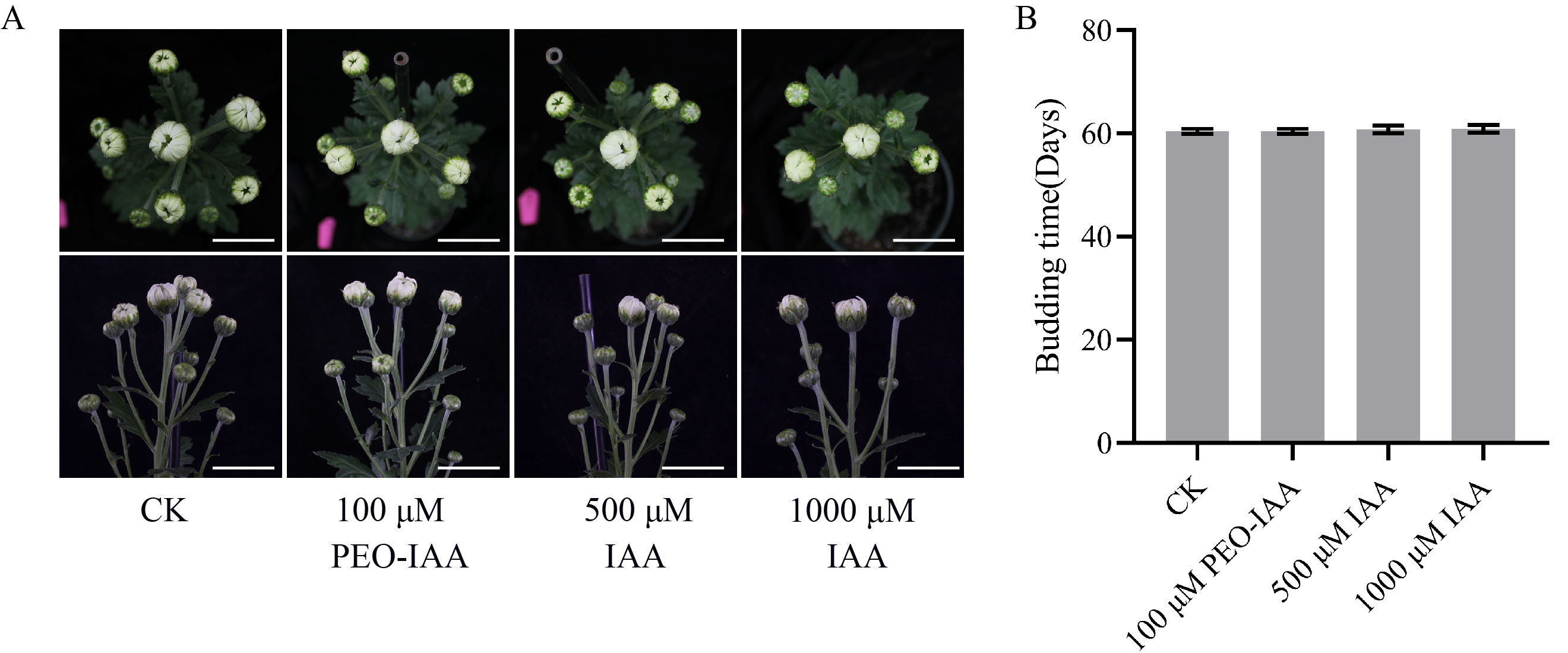


**Figure S11.** Effects of auxin and auxin antagonist treatments on flowering time in chrysanthemum ‘Jinba’. (A) Phenotypic comparison of flowering time in chrysanthemum under auxin and auxin antagonist treatments. (B) Statistical analysis of flowering time under different treatments. The values are presented as the mean ± standard deviation (n = 15).
