# Supplementary material for: CmARF3–CmTCP7 module regulates flowering time in chrysanthemum (Chrysanthemum morifolium)
Source: Hortic Res. 2025 May 23;12(7):uhaf095. doi: 10.1093/hr/uhaf095 (PMC12099206; doi:10.1093/hr/uhaf095)
Supplement: Web_Material_uhaf095 [file web_material_uhaf095.zip › Supplemental_Tables_uhaf095.docx]

**Supplemental Tab. S1 Analysis of *cis*-elements in CmTCP7 promoter region.**

| **Element** | **Function** | **Location** |
| --- | --- | --- |
| ACGTA BOX (TACGTA) | Sugar accumulation response inhibition | 910 (+) 910 (-) |
| CAAT BOX (CCAAT) | Common cis-acting element in promoter and enhancer regions | 1231 (+) |
| GATA BOX (GATA) | CaMV 35S promoter | 292 (+) 614 (+) 817 (+) 862 (+) 150 (-) 399 (-) 600 (-) 804 (-) 1287 (-) |
| I BOX (GATAAG) | Part of a light responsive element | 397 (-) 1285 (-) |
| TATA BOX2 (TATAAAT) | Position the combination of RNA polymerase | 557 (+) 942 (+) 924 (-) 939 (-) |
| W BOXNTERF3 (TGACY) | Damage response activation ERF3 genes | 649 (+) 574 (-) |
| ***** **AuxRE (TGTCTC)** | **ARF(Auxin response factor) binding domain** | **633 (+) 1208 (+) 550 (-)** |
| ARR1AT (NGATT) | ARR1 binding domain | 28 (+) 1053 (+) 269 (-) 329 (-) 368 (-) 405 (-) 416 (-) 442 (-) 538 (-) 694 (-) 704 (-) 789 (-) 871 (-) 890 (-) 1036 (-) 1072 (-) 1232 (-) |
| *CATATGGMSAUR (CATATG) | Involved in auxin response | 408 (+) 408 (-) |
| CPBCSPOR (TATTAG) | Participate in cytokinins enhanced protein binding | 482 (-) |
| GAREAT (TAACAAR) | Related to the GA response and induce | 973 (+) |
| MYB1AT (WAACCA) | Related to the ABA signal | 854 (+) 1025 (+) 263 (+) 362 (+) |
| MYBGAHV (TAACAAA) | Core of GA response complex GARC | 973 (+) |
| *NTBBF1ARROLB (ACTTTA) | Involved in auxin response | 811 (+) 163 (-) |
| POLASIG (AATAAA/T) | Participate in the polyA signal | 946 (+) 1022 (+) 997 (-) 439 (+) 1100 (+) 827 (-) |
| *SURECOREATSULTR11 (GAGAC) | ARF combined sequence | 550 (+) 634 (-) 663 (-) 1209 (-) |

**Supplemental Tab. S2 The yeast one-hybrid library analysis by three tandem copies of TGTCTC**

| H1-10 | PREDICTED: 40S ribosomal protein S25 |
| --- | --- |
| H1-13 | acyl carrier protein 2 |
| H1-15 | PREDICTED: photosystem I reaction center subunit psaK, chloroplastic |
| H1-11 | Ribosomal protein L37 |
| H1-18 | PREDICTED: uncharacterized protein At4g22758-like |
| H1-19 | chloroplast light-harvesting chlorophyll a/b-binding protein |
| H1-20 | PREDICTED: thiamine thiazole synthase 1, chloroplastic |
| H1-21 | PREDICTED: histone H3.2-like |
| H1-28 | PREDICTED: ribosomal protein S11, mitochondrial |
| H1-29 | PREDICTED: uncharacterized protein LOC105050026 isoform X1 |
| H1-30 | PREDICTED: cytochrome b-c1 complex subunit 6-like |
| **H1-32** | **PREDICTED: auxin response factor** |
| H2-2-4 | 40S ribosomal S13 |
| H2-2-5 | PREDICTED: photosystem II 10 kDa polypeptide, chloroplastic |
| H2-2-6 | farnesyl diphosphate synthase |
| H2-2-10 | PREDICTED: serine/arginine-rich SC35-like splicing factor SCL30 isoform X1 |
| H2-2-11 | phosphatase 2C family protein |
| H2-2-13 | PREDICTED: transcription factor RF2b isoform X2 |
| H2-2-16 | PREDICTED: abscisic acid receptor PYL8-like isoform X2 |
| H2-2-18 | ribulose-1,5-bisphosphate carboxylase small subunit |
| H2-2-19 | chloroplast light-harvesting chlorophyll a/b-binding protein |
| H2-2-25 | Ubiquitin supergroup, Ribosomal protein L40e |
| H2-2-26 | PREDICTED: 40S ribosomal protein S27-2 |
| H2-2-29 | PREDICTED: 40S ribosomal protein S16-like |
| H2-2-31 | PREDICTED: heavy metal-associated isoprenylated plant protein 26-like |
| H2-2-37 | PREDICTED: 40S ribosomal protein S19-1-like |
| H2-2-76 | thaumatin-like protein |

**Supplemental Tab. S3 The oligos used in this study**

| Name | Sequence (5' to 3') | Note |
| --- | --- | --- |
| Kan-F | TCTGATGCCGCCGTGTTC | PCR |
| Kan-R | GATGTTTCGCTTGGTGGTCG | PCR |
| 35S-F | AGATACAGTCTCAGAAGACCAAAGG | PCR |
| 35S-R | TTGATATTCTTGGAGTAGACGAGAG | PCR |
| CmTCP7-3-GSP1 | ACAACTGACGGCACAACTAACG | 3’RACE |
| CmTCP7-3-GSP2 | TACTGTTAGCGTTGCTGTTAGGTCC | 3’RACE |
| CmTCP7-3-GSP3 | GAGATTGATAAAGATGATGGGCAGAG | 3’RACE |
| CmTCP7-5-GSP1 | CTGCTGATAAAACCTCCCTTGC | 5’RACE |
| CmTCP7-5-GSP2 | ACGCCCAAAACCCACCTC | 5’RACE |
| CmTCP7-5-GSP3 | TCATCCAACCTTAACCTCTTCCC | 5’RACE |
| CmTCP7-fulllength-F | ATGTCAACAACTGACGGCACAACTA | Clone |
| CmTCP7-fulllength-R | CAACAAACAGCGCCAGATAAATAAA | Clone |
| CmFDL1-fulllength-F | ATGAAACCAACAGATGACGTATGG | Clone |
| CmFDL1-fulllength-R | AAATGGGGCACTTTTGGTTCTGTG | Clone |
| CmARF3-3-GSP1 | TAATCGTCCCTCAGAAACCAC | 3’RACE |
| CmARF3-3-GSP2 | TCGTGCCATAAACATTACCAA | 3’RACE |
| CmARF3-3-GSP3 | TTGTTGGGAGTTTCTGATTGC | 3’RACE |
| CmARF3-5-GSP1 | AACAGTCCTCTGCTGCTCTACG | 5’RACE |
| CmARF3-5-GSP2 | GCCTTGTGGGAAGTAAACGAC | 5’RACE |
| CmARF3-5-GSP3 | CCACTAATGAACCCGACTTTG | 5’RACE |
| CmARF3-fulllength-F | CTGGATTCGCCTACTTCT | Clone |
| CmARF3-fulllength-R | CAGAAACTCCCAACAACATAC | Clone |
| CmTCP7-anti-pBIG-F | CGAGCTCATGTCAACAACTGACGGCAC | Transformation |
| CmTCP7-anti-pBIG-R | GCTCTAGACAGCGCCAGATAAATAAATTC | Transformation |
| Oligos for amiRNA | TCAGTAGTCAGTAAATGGCTC | Transformation |
| I miR-s | gaTCAGTAGTCAGTAAATGGCTCtctctcttttgtattcc | Transformation |
| II miR-a | gaGAGCCATTTACTGACTACTGAtcaaagagaatcaatga | Transformation |
| III miR*s | gaGAACCATTTACTGTCTACTGTtcacaggtcgtgatatg | Transformation |
| IV miR*a | gaACAGTAGACAGTAAATGGTTCtctacatatatattcct | Transformation |
| CmEF1α-F | TTTTGGTATCTGGTCCTGGAG | Real-time PCR |
| CmEF1α-R | CCATTCAAGCGACAGACTCA | Real-time PCR |
| CmTCP7-Q-RTF | CAGCAAGGGAGGTTTTATCAGC | Real-time PCR |
| CmTCP7-Q-RTR | GATATCAACAAACAGCGCCAGA | Real-time PCR |
| CmARF3-Q-RTF | GCGATCTCAGGAACCTCATTCACAT | Real-time PCR |
| CmARF3-Q-RTR | GTAGAACGGATGCAGGCGAGTG | Real-time PCR |
| CmCDM111L-Q-RTF | GAAAGATGGGAAGAGGTAAGGT | Real-time PCR |
| CmCDM111L-Q-RTR | GGCTTTCTTCAATAAACCACC | Real-time PCR |
| CmFTL3-Q-RTF | GGGAAGTTGCTAACGGGTGTGAG | Real-time PCR |
| CmFTL3-Q-RTR | CCTGTTGTCGCTGGAATATCGGTAA | Real-time PCR |
| EMSAARF3-TCP7P-1 | AGTGCATTGAGGTAGACAATCGGTATAACGAGACATTATAAATATTAAAACCTGGTCAG | EMSA |
| EMSAARF3-TCP7P-2 | TTAACGCAAGGATATACGCCCACATGTCATGTCTCCTCTATGGGATGACCCTCACGTGG | EMSA |
| EMSAARF3-TCP7P-3 | ATATATGGCCCACCCACCCATGTGCCCTCCTGTCTCCACCAGCTCCTGCCGCTCAATCA | EMSA |
| EMSAARF3-TCP7P-4 | AGACAATCGGTATAACGAGACATTATAAATATTAAAACCTGGTCAGTAAAACCTGCATCACTCTTATATCTTAACGCAAGGATATACGCCCACATGTCATGTCTCCTCTATGGGATGACC | EMSA |
| EMSAARF3-TCP7P-MCP1 | AGTGCATTGAGGTAGACAATCGGTATAACGAtcCATTATAAATATTAAAACCTGGTCAG | EMSA |
| EMSAARF3-TCP7P-MCP2 | TTAACGCAAGGATATACGCCCACATGTCATGgaTCCTCTATGGGATGACCCTCACGTGG | EMSA |
| EMSAARF3-TCP7P-MCP3 | ATATATGGCCCACCCACCCATGTGCCCTCCTGgaTCCACCAGCTCCTGCCGCTCAATCA | EMSA |
| EMSAARF3-TCP7P-MCP-41 | AGACAATCGGTATAACGAtcCATTATAAATATTAAAACCTGGTCAGTAAAACCTGCATCACTCTTATATCTTAACGCAAGGATATACGCCCACATGTCATGTCTCCTCTATGGGATGACC | EMSA |
| EMSAARF3-TCP7P-MCP-42 | AGACAATCGGTATAACGAGACATTATAAATATTAAAACCTGGTCAGTAAAACCTGCATCACTCTTATATCTTAACGCAAGGATATACGCCCACATGTCATGgaTCCTCTATGGGATGACC | EMSA |
| EMSAARF3-TCP7P-MCP-412 | AGACAATCGGTATAACGAtcCATTATAAATATTAAAACCTGGTCAGTAAAACCTGCATCACTCTTATATCTTAACGCAAGGATATACGCCCACATGTCATGgaTCCTCTATGGGATGACC | EMSA |
| CHIP-TCP7Pro-2F | GGTAGACAATCGGTATAACGAGACA | CHIPQPCR |
| CHIP-TCP7Pro-2R | CGTGAGGGTCATCCCATAGAGG | CHIPQPCR |
| CHIP-TCP7Pro-3F | ACATAGCTCTTGGGACGACCTC | CHIPQPCR |
| CHIP-TCP7Pro-3R | TGTCTCGTTATACCGATTGTCTACC | CHIPQPCR |
| CHIP-TCP7Pro-4F | TTAACGCAAGGATATACGCCCACAT | CHIPQPCR |
| CHIP-TCP7Pro-4R | GTGCTTGTTCGCTTCTCATGGAATG | CHIPQPCR |
| CHIP-TCP7Pro-5F | CCATGAGAAGCGAACAAGCACATC | CHIPQPCR |
| CHIP-TCP7Pro-5R | CGTAAAGCATTGATTAGTGAAATGAAAGGA | CHIPQPCR |
| CHIP-TCP7Pro-6F | GGAAGGTTAGACACACTCGTGAATG | CHIPQPCR |
| CHIP-TCP7Pro-6R | AGTCTAATGATTGAGCGGCAGGAG | CHIPQPCR |
| CHIP-TCP7ORF-7F | TCGGCGGCTAGGGTTGGGAATT | CHIPQPCR |
| CHIP-TCP7ORF-7R | TGCCTCCTCCAGAAGACTGCTGAG | CHIPQPCR |
| MCS-F1 | GCTAGCGGCCGGCCGAATTCACGCGTAAGCTTGAGCTCATCGA | Construction |
| MCS-F2 | TAAGCTTGAGCTCATCGATACCGGTTCTAGAGCGATCGCCTGC | Construction |
| MCS-F3 | TCTAGAGCGATCGCCTGCAGCTCGAGCGCCGGCGGCTAGCGGATCC | Construction |
| CmTCP7pro-F | AGCTTTCTCGTGTTTAGCAACTTTG | Clone |
| CmTCP7pro-R | GGTTGTTTGTGTGTGTGAAAAGAGT | Clone |
| CmCDM111Lpro-F | ACATCAACCATGCTAAACAGCTTCTC | Clone |
| CmCDM111Lpro-R | CACTAATGAACATCCAACACCCTTGT | Clone |
| A-Xba-PCVA | ctcagatctggatcctctagaCTGCAAGGCGATTAAGTTGGG | Construction |
| B-Kpn-PCVA | cctaagcggctagcggtaccGCGGATAACAATTTCACACAGGA | Construction |
| CmTCP7-I | gaTTAATAGGCATGCGTACCCTCtctctcttttgtattcc | Construction |
| CmTCP7-II | gaGAGGGTACGCATGCCTATTAAtcaaagagaatcaatga | Construction |
| CmTCP7-III | gaGAAGGTACGCATGGCTATTATtcacaggtcgtgatatg | Construction |
| CmTCP7-IV | gaATAATAGCCATGCGTACCTTCtctacatatatattcct. | Construction |

**Supplemental Tab. S4 Analysis of *cis*-elements (TCP family binding sites) in CmCDM111L promoter region.**

| Matrix ID | Family | Position | Strand | Similar Score | Hit Sequence |
| --- | --- | --- | --- | --- | --- |
| TFmatrixID_0422 | TCP | -1581 | - | 0.93 | actGGTCCaa |
| TFmatrixID_0440 | TCP | -1580 | - | 0.93 | ctGGTCCa |
| TF_motif_seq_0266 | TCP | -1578 | + | 1 | GGTCC |
| TF_motif_seq_0266 | TCP | -1578 | - | 1 | GGTCC |
| TF_motif_seq_0431 | TCP | -1578 | + | 0.75 | GGTCCaaa |
| TF_motif_seq_0266 | TCP | -1570 | + | 0.75 | GATCC |
| TF_motif_seq_0266 | TCP | -1570 | - | 0.75 | GATCC |
| TF_motif_seq_0266 | TCP | -1556 | + | 0.75 | GGTAC |
| TF_motif_seq_0266 | TCP | -1556 | - | 0.75 | GGTAC |
| TF_motif_seq_0431 | TCP | -1556 | + | 0.75 | GGTACaac |
| TF_motif_seq_0266 | TCP | -1541 | + | 0.75 | GGTCT |
| TF_motif_seq_0266 | TCP | -1541 | - | 0.75 | GGTCT |
| TF_motif_seq_0266 | TCP | -1527 | + | 0.75 | GGTTC |
| TF_motif_seq_0266 | TCP | -1527 | - | 0.75 | GGTTC |
| TF_motif_seq_0266 | TCP | -1514 | + | 0.75 | GGAGC |
| TF_motif_seq_0266 | TCP | -1514 | - | 0.75 | GGAGC |
| TF_motif_seq_0266 | TCP | -1469 | + | 0.75 | GGGTC |
| TF_motif_seq_0266 | TCP | -1469 | - | 0.75 | GGGTC |
| TF_motif_seq_0266 | TCP | -1468 | + | 0.75 | GGTCA |
| TF_motif_seq_0266 | TCP | -1468 | - | 0.75 | GGTCA |
| TF_motif_seq_0266 | TCP | -1408 | + | 0.75 | GGTCA |
| TF_motif_seq_0266 | TCP | -1408 | - | 0.75 | GGTCA |
| TF_motif_seq_0266 | TCP | -1403 | + | 0.75 | GGTTC |
| TF_motif_seq_0266 | TCP | -1403 | - | 0.75 | GGTTC |
| TF_motif_seq_0431 | TCP | -1403 | + | 0.75 | GGTTCaac |
| TF_motif_seq_0266 | TCP | -1370 | + | 0.75 | GGACT |
| TF_motif_seq_0266 | TCP | -1370 | - | 0.75 | GGACT |
| TF_motif_seq_0266 | TCP | -1362 | + | 0.75 | GGACA |
| TF_motif_seq_0266 | TCP | -1362 | - | 0.75 | GGACA |
| TF_motif_seq_0266 | TCP | -1353 | + | 0.75 | GGTCG |
| TF_motif_seq_0266 | TCP | -1353 | - | 0.75 | GGTCG |
| TF_motif_seq_0266 | TCP | -1340 | + | 0.75 | GGTCA |
| TF_motif_seq_0266 | TCP | -1340 | - | 0.75 | GGTCA |
| TF_motif_seq_0266 | TCP | -1248 | + | 0.75 | GGATC |
| TF_motif_seq_0266 | TCP | -1248 | - | 0.75 | GGATC |
| TF_motif_seq_0266 | TCP | -1242 | + | 0.75 | GGTAC |
| TF_motif_seq_0266 | TCP | -1242 | - | 0.75 | GGTAC |
| TF_motif_seq_0266 | TCP | -1218 | + | 0.75 | GCTCC |
| TF_motif_seq_0266 | TCP | -1218 | - | 0.75 | GCTCC |
| TF_motif_seq_0266 | TCP | -1072 | + | 0.75 | GGTCT |
| TF_motif_seq_0266 | TCP | -1072 | - | 0.75 | GGTCT |
| TF_motif_seq_0266 | TCP | -1008 | + | 0.75 | GGTTC |
| TF_motif_seq_0266 | TCP | -1008 | - | 0.75 | GGTTC |
| TF_motif_seq_0266 | TCP | -874 | + | 0.75 | AGTCC |
| TF_motif_seq_0266 | TCP | -874 | - | 0.75 | AGTCC |
| TF_motif_seq_0431 | TCP | -874 | + | 0.75 | AGTCCcgc |
| TF_motif_seq_0266 | TCP | -873 | + | 0.75 | GTCCC |
| TF_motif_seq_0266 | TCP | -873 | - | 0.75 | GTCCC |
| TF_motif_seq_0266 | TCP | -744 | + | 0.75 | GTTCC |
| TF_motif_seq_0266 | TCP | -744 | - | 0.75 | GTTCC |
| TF_motif_seq_0266 | TCP | -732 | + | 0.75 | GGCAC |
| TF_motif_seq_0266 | TCP | -732 | - | 0.75 | GGCAC |
| TF_motif_seq_0266 | TCP | -692 | + | 0.75 | GGTCG |
| TF_motif_seq_0266 | TCP | -692 | - | 0.75 | GGTCG |
| TF_motif_seq_0266 | TCP | -579 | + | 0.75 | GGTCG |
| TF_motif_seq_0266 | TCP | -579 | - | 0.75 | GGTCG |
| TF_motif_seq_0266 | TCP | -568 | + | 0.75 | TGACC |
| TF_motif_seq_0266 | TCP | -568 | - | 0.75 | TGACC |
| TF_motif_seq_0266 | TCP | -369 | + | 0.75 | GGACA |
| TF_motif_seq_0266 | TCP | -369 | - | 0.75 | GGACA |
| TF_motif_seq_0266 | TCP | -363 | + | 0.75 | GTACC |
| TF_motif_seq_0266 | TCP | -363 | - | 0.75 | GTACC |
| TF_motif_seq_0431 | TCP | -251 | - | 0.75 | gttGTACC |
| TF_motif_seq_0266 | TCP | -248 | + | 0.75 | GTACC |
| TF_motif_seq_0266 | TCP | -248 | - | 0.75 | GTACC |
| TF_motif_seq_0266 | TCP | -143 | + | 0.75 | AGACC |
| TF_motif_seq_0266 | TCP | -143 | - | 0.75 | AGACC |
| TF_motif_seq_0266 | TCP | -103 | + | 0.75 | GGAAC |
| TF_motif_seq_0266 | TCP | -103 | - | 0.75 | GGAAC |
| TF_motif_seq_0431 | TCP | -88 | - | 0.75 | ttgGGACA |
| TF_motif_seq_0266 | TCP | -86 | + | 0.75 | GGGAC |
| TF_motif_seq_0266 | TCP | -86 | - | 0.75 | GGGAC |
| TF_motif_seq_0266 | TCP | -85 | + | 0.75 | GGACA |
| TF_motif_seq_0266 | TCP | -85 | - | 0.75 | GGACA |

**Supplemental Tab. S5** Expression levels of *CmTCP* genes in WT and *antiCmTCP7* lines

| Genotype | *CmTCP8*(FPKM) | *CmTCP21*(FPKM) | *CmTCP22*(FPKM) | *CmTCP23*(FPKM) |
| --- | --- | --- | --- | --- |
| WT | 10.61 | 50.78 | 12.24 | 30.93 |
| *AntiCmTCP7-3* | 8.58 | 51.14 | 11.6 | 32.51 |
| *AntiCmTCP7-4* | 10.55 | 57.06 | 12.13 | 41.27 |
